# Multilabel user classification using the community structure of online networks

**DOI:** 10.1371/journal.pone.0173347

**Published:** 2017-03-09

**Authors:** Georgios Rizos, Symeon Papadopoulos, Yiannis Kompatsiaris

**Affiliations:** Information Technologies Institute, CERTH, Thermi Thessaloniki, Greece; Nanjing Normal University, CHINA

## Abstract

We study the problem of semi-supervised, multi-label user classification of networked data in the online social platform setting. We propose a framework that combines unsupervised community extraction and supervised, community-based feature weighting before training a classifier. We introduce Approximate Regularized Commute-Time Embedding (ARCTE), an algorithm that projects the users of a social graph onto a latent space, but instead of packing the global structure into a matrix of predefined rank, as many spectral and neural representation learning methods do, it extracts local communities for all users in the graph in order to learn a sparse embedding. To this end, we employ an improvement of personalized PageRank algorithms for searching locally in each user’s graph structure. Then, we perform supervised community feature weighting in order to boost the importance of highly predictive communities. We assess our method performance on the problem of user classification by performing an extensive comparative study among various recent methods based on graph embeddings. The comparison shows that ARCTE significantly outperforms the competition in almost all cases, achieving up to 35% relative improvement compared to the second best competing method in terms of F1-score.

## Introduction

User classification in Online Social Networks (OSNs) is the problem of inferring the interests, associated themes, expertise and other attributes of a user based on their online behavior. It has successfully found application in personalized content and user recommendation [1], targeted advertising and marketing, expert search and monitoring [2] and social search and promotion [3, 4]. A more recent application is the automated extraction of information sources, i.e. users and communities that are relevant to news stories [5] and the identification and tracking of experts by journalists given an initial seed set [6]. Finally, a recent interesting application pertains to the behavioral analysis of online user communities that partake in political discussions based on identifying the political affiliation of users [7] or communities/blogs [8].

The need for improved automated classification of public OSN user profiles is motivated by the lack of reliable user annotation. The majority of online accounts do not explicitly publish their interests, leading to sparsely and noisily labeled datasets. Specifically, the textual self-descriptions that users provide in their online profiles are often too generic to be of any value, false, inaccurate, or simply unavailable. Furthermore, large-scale annotation by experts is unfeasible due to the large amount of effort that would be required for such a task. Yet, the large amount of social connections, interactions and messages by online users, which are publicly visible and can be automatically collected and analyzed, could be leveraged as informative signals for user classification.

Out of such informative signals, text messages may seem to be the most obvious choice. However, such signals often prove to be noisy or insufficient due to brevity (e.g., in Twitter), ambiguity or multi-linguality. This has been shown to be an obstacle in short text clustering [9]. A different type of signal relies on the structure of user graphs and is based on the principle of *homophily* [10]; i.e. people sharing the same beliefs and interests tend to connect to each other and are expected to form denser than average *communities*. For example, topic experts on Twitter are very often located within one hop from each other [11]. The homophily principle may be leveraged from a computational point of view by the *manifold* assumption of semi-supervised learning. The latter states that the classification for adjacent samples should be smooth. The appeal of this approach is that it exposes one more facet of this graph-based problem to analysis via the rich relevant literature while being complementary to content-based analysis.

**Fig 1:**
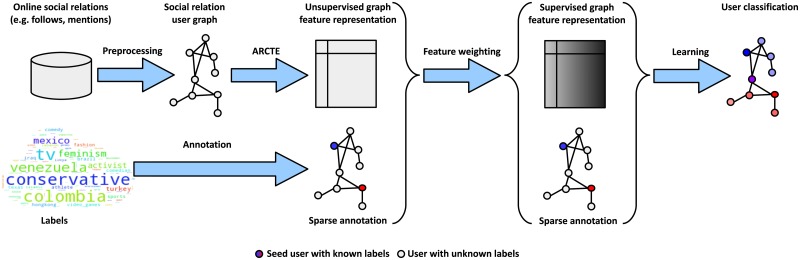
Overview of community-based user classification framework.

The *social dimension* approach to graph-based, semi-supervised learning aims to avoid costly time and space consuming matrix inversions [12] and learning label-dependent hypotheses [13] by first embedding the graph in an unsupervised way on a latent space and then using the coordinates as features for training a classifier. To our knowledge, no previous attempt has been made to generate detailed representations of the structure of the users’ social graph for user classification by embedding a graph that captures the structural information contained in user-centric communities from the standpoint of each user. In particular, our contributions to multi-label, semi-supervised user classification by means of learning graph representations are the following:

- We introduce user-centric community detection for graph-based user classification, as a means to capture, for *every* user in a graph, missing links to similar, but not directly connected users. To this end, we propose an algorithm that extracts *user-centric communities* in a scalable and highly parallelizable way, and uses them to embed the user graph in a latent feature space. We name our method Approximate Regularized Commute-Time Embedding (ARCTE).
- As an additional benefit, we propose two improvements upon known methods for calculating user-centric PageRank vectors to be used in user-centric community detection: *a*) we derive a method to calculate cumulative PageRank differences, a more potent similarity measure for local graph exploration, *b*) we reduce the number of required operations caused by unnecessary user self-connections in user-centric methods.
- We extend the proposed community-centric user representation by introducing a supervised feature weighting step that boosts the importance of communities that include similarly annotated users with statistical significance. An overview of the proposed *community-based embedding framework* is illustrated in Fig 1.
- We conduct an extensive comparative study of numerous feature extraction methods in a multi-label user classification task. The results indicate that standard community detection techniques do not manage to extract informative features for user classification. Furthermore, ARCTE surpasses several recent spectral and deep representation learning approaches and also achieves one of the most favorable accuracy-complexity trade-offs compared to the competition.
- Finally, we also introduce a new dataset (SNOW 2014 Graph) for multi-label user classification, on which we show the results of graph-based user classification leveraging social interactions (e.g., mentions, retweets) on a Twitter stream sample collected during the course of a day.

We provide an implementation of ARCTE and the SNOW 2014 Graph dataset at the project’s GitHub page (https://github.com/MKLab-ITI/reveal-graph-embedding).

## Graph-based user classification

### Background

We model user classification as a *multi-label, graph-based, semi-supervised classification problem*, in which the input data do not inhabit a Euclidean space but are described via their pairwise relations. Let *G* = (*V*, *E*) be the graph (i.e. network) of users, where *V* is the set of vertices (i.e. users) and *E* the set of edges (i.e. relations). The graph is represented by an adjacency matrix *A*, where *A*(*u*, *v*) = *a*_*uv*_, ∀*u*, *v* ∈ *E* is the directed edge weight from user *u* to user *v*. In this study we discuss undirected graphs, in which the adjacency matrix is symmetric (*A*′ = *A*). We will use the colon notation *A*_*u*:_, *A*_:*v*_ to symbolize a single matrix row or column respectively. We further denote by *N*(*v*) the set of neighbors of vertex *v* and by *d*(*v*) its degree. Let *L* be the set of labels that characterize users. Let *V*_*l*_ be the labeled and *V*_*u*_ the unlabeled user set. For each user *u* ∈ *V*, let *y*_*v*_ be the labeling vector, that contains 1 if user *v* is annotated with the corresponding label and 0 otherwise. We denote by *Y* the label matrix, where each row *v* corresponds to the labeling vector of user *v*. Finally, we define the matrices *Y*_*l*_ and *Y*_*u*_ that correspond to the sets *V*_*l*_ and *V*_*u*_.

Given the above notation, user classification is the problem of inferring the labels of the unlabelled set *Y*_*u*_ given the adjacency matrix *A* and the known labels *Y*_*l*_. However, in a real-world scenario we may get more annotated users or more labels for an already annotated user. This may either be the result of additional expert input, or an automated process for annotation. In such cases, it is desirable to have the bulk of the computational work be label-independent. This can be done by performing a feature extraction step on the graph in order to project the graph vertices to a latent Euclidean space such that vertex proximity is preserved. This has been termed the *social dimension* approach [14]. The user coordinates in this latent space are denoted by the user feature matrix *X*. The coordinates of vertex *v* are denoted by *X*_*v*:_ ∈ ℜ^*dim*^, where *dim* is the dimensionality of the latent space. Subsequently we also have: *X*_*l*_ and *X*_*u*_. The latent space features are label-independent and as such reusable even when new labels become available. The framework qualifies as semi-supervised learning regardless of whether a supervised or semi-supervised approach is selected to train a *hypothesis* *h* that maps user projections in *X* to label vectors in *Y*, because the full graph is used to extract *X*. To deal with the multi-label nature of the problem, any multi-label classification scheme may be used [15], such as One-vs-All. The above process is formalized in Alg 1.

**Algorithm 1** User Classification Framework

INPUT: *A*, *V*_*l*_, *V*_*u*_, *Y*_*l*_ and *dim*

OUTPUT: *Y*_*u*_

1: $X=\left\{ \begin{array}{l} \text{low-rank\_embedding}\left(A,dim\right)\\ \text{community\_embedding}\left(A,dim\right) \end{array}\right.$         ▷ Graph embedding

2: $h=\left\{ \begin{array}{l} \text{multi-label}\left(\text{supervised\_classifier}\left(X_{l},Y_{l}\right)\right)\\ \text{multi-label}\left(\text{semi-supervised\_classifier}\left(X_{l},X_{u},Y_{l}\right)\right) \end{array}\right.$   ▷ Hypothesis

3: *Y*_*u*_ = *h*(*X*_*u*_)                    ▷ Out-of-sample prediction

### Limitations of existing graph embedding approaches

**Fig 2:**
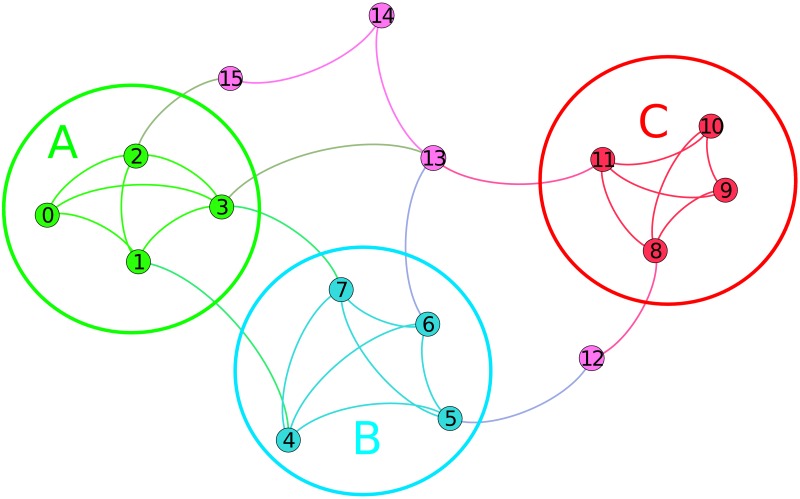
A toy graph.

We now describe the strengths and weaknesses of various graph embedding approaches with the help of the toy graph depicted in Fig 2. We assume the presence of three labels (A, B, C) and hypothesize that users with common labels give rise to dense social communities due to *homophily*. Furthermore, there exist inter-community edges that imply the existence of boundary vertices with multiple affiliations. To complicate matters further, there exist other vertices with no clear affiliations.

#### Low-rank matrix embedding

Many existing approaches for embedding graphs attempt to fit the full graph representation into a latent space of a small, pre-defined number of dimensions *dim*. Their motivation is that a low dimensional projection leads to improved generalization and that naturally continuous features as produced by spectral (e.g., Modularity Maximization as in [14], Laplacian Eigenmaps as in [16], RWModMax [17]) or neural representation learning (e.g., DeepWalk [18], LINE [19]) approaches better capture the affiliation degree of a vertex to latent social dimensions. However, this may lead to only a “rough” representation that would lack a lot of structural information given that the dimensionality of the feature matrix is selected a-priori. The alternative is to use expensive cross-validation and fine-tune the dimensionality to a specific labeling. Furthermore, spectral methods calculate a number of eigenvectors equal to the dimensionality *dim*, something that can be very costly. It must also be noted that when the graph size is very large, the space required to store the features (*O*(*dim* ⋅ |*V*|)) becomes a non-trivial problem [20].

We run Laplacian Eigenmaps [21] on our toy graph, shown in section A of Table 1. We show the first three non-trivial eigenvectors (*dim*_1–3_) of the graph Laplacian matrix, which are sufficient to capture the community structure of this simple graph. We note that vertices 9 and 10 are embedded at the same point in *X* due to structural equivalence.

**Table 1:** Examples of low-rank and community-based representations.

**A**				
	*dim*_1_	*dim*_2_	*dim*_3_	
*v*_4_	0.151	−0.342	−0.024	
*v*_12_	−0.146	−0.198	−0.141	
*v*_13_	0.019	0.09	0.362	
*v*_15_	0.155	0.334	0.394	
**B**				
	*A*_*d*_	*B*_*d*_	*C*_*d*_	
*v*_4_	0	1	0	
*v*_12_	0	0	1	
*v*_13_	1	0	0	
*v*_15_	1	0	0	
**C**				
	*A*_*o*_	*B*_*o*_	*C*_*o*_	
*v*_4_	0	1	1	
*v*_12_	0	1	1	
*v*_13_	1	1	0	
*v*_15_	1	0	0	
**D**				
	*A*	*B*	*C*	*AB*
*v*_4_	0	1	0	1
*v*_12_	0	0	1	0
*v*_13_	0	0	1	0
*v*_15_	1	0	0	0

**A**: Spectral, **B**: Disjoint, **C**: Overlapping and **D**: Hierarchical.

#### Community-based embedding

Attempting to address some of the limitations of low-rank embedding methods, a limited number of approaches using community detection [22] have been tried for user embedding. These include the overlapping EdgeCluster method [20] and the hierarchical Multi-Resolution Overlapping Communities (MROC) method [23]. We first explain the means by which the community-based embedding is made and then discuss the theoretical limitations of typical community detection paradigms used for its formation. Suppose we execute any community detection method. The number of communities detected is |*C*|, where *C* is the full set of communities. The number of vertices in a community *c* is |*c*|. Graph vertices are then represented by the community indicator matrix *X*, defined in Eq 1.

$$\begin{array}{c} x_{vd}=\left\{\begin{array}{cc} 1, & \text{if}\;v\in c_{d}\\ 0, & \text{otherwise} \end{array}\right.,\forall v\in V,c_{d}\in C, \end{array}$$ (1)

We call this operation *community-based embedding*, denoted by *X* = embed(*C*). Another way to view such an embedding is a binary representation such as those usually found in text classification where the communities are terms, the vertices are documents and the non-zero values of *X* denote a document containing a term. By aggregating a number of vertices into a community, one implicitly encodes a kind of similarity between the vertices of the same community, even though they may not be directly linked. The community-based embedding examples in sections B-D of Table 1 correspond to three main types of community: disjoint, overlapping and hierarchical.

**Disjoint community detection:** Given Eq 1, each vertex embedding will have exactly one non-zero element in the dimension corresponding to the community it belongs. More importantly, all the vertices belonging to the same community are collocated at the same point on *X*. For example, suppose that a hypothetical disjoint method correctly groups together vertices 0–3 as community *A*_*d*_, vertices 4–7 as *B*_*d*_ and vertices 8–11 as *C*_*d*_ (see section B of Table 1). Now suppose that vertex 12 gets allocated to community *C*_*d*_. That would mean it would be on the same latent point as all vertices from label C, whereas it is highly likely that this vertex is also associated with label B.

**Overlapping community detection:** This type of community leads to higher distinctiveness in vertex embeddings on *X* by possibly associating a vertex with multiple latent dimensions. Suppose now that a hypothetical overlapping method correctly groups together vertices 0–3 as community *A*_*o*_, vertices 4–7 as *B*_*o*_ and vertices 8–11 as *C*_*o*_ (see section C of Table 1). It *might* be possible for such a method to have vertex 12 also be assigned to *B*_*o*_ and *C*_*o*_.

**Hierarchical community detection:** In such cases, vertices are embedded in *X* using their assignment to communities at different resolutions. If we consider vertex 4 to be a boundary vertex associated both with label A and label B, then a hypothetical hierarchical scheme that considers a hyper-community comprising vertices both of label A and B (*AB*—see section D of Table 1) after merging communities A and B, will capture the multiple affiliation successfully. Still, all the other vertices from label B will also share a common feature with all the label A ones, something that may be undesired in case there is no true label hierarchy.

#### Vertex-centric community embedding

We posit that since each vertex has its own local view of the graph, considering vertex-centric communities results in a vertex representation that is much more granular and detailed compared to the previously presented community representations. We now describe *two types of vertex-centric community, the first capturing the local connectivity structure in high resolution, and the second additionally capturing missing links between vertices*.

**Base vertex-centric communities:** We define the very high resolution *base community* [23]*b*_*v*_ of a vertex *v* as the set of adjacent vertices plus the ego vertex, *b*_*v*_ = *N*(*v*) ∪ *v*. Their value as high resolution features that encode user preferences can be understood by considering vertex 13. Many techniques that aim for a mesoscopic community representation of a graph might overlook the possible triple affiliation implied for vertex 13 and there is no guarantee that they will capture it. Furthermore, hierarchical community detection might not be of use in this case, as the affiliation of this vertex to a hyper-community containing numerous (or all) vertices is not very informative. In contrast, the base community of a vertex implies similarity among its member vertices, as it captures vertex-centric preferences given that the connections were made voluntarily (see *homophily*). The same applies to vertices 12 and 4. Consider the feature matrix *X*_*base*_ = embed(*B*), where *B* is the set of all base communities. Using base communities for vertex embeddings results in points in the high-dimensional space ℜ^|*V*|^, which are by definition proximal to the ones of adjacent vertices. However, in sparsely annotated graphs many vertices and especially the ones with small degree, will not have annotated neighbors and thus will be difficult to classify using just base communities.

**Extended vertex-centric communities:** In order to capture missing links for each vertex in the graph, we propose the inclusion of an additional type of community that is based on *local searches around each vertex*. We consider *extended vertex-centric communities* *e*_*v*_ that contain the neighbors of a vertex plus any not directly connected, yet similar vertices. To this end, we will use a type of random walk based similarity called regularized commute-times, to be described in sub-sub-section *Fast similarity vector Calculation*. See, for example, in section B of Table 2 that the local community around vertex 13 (*e*_13_) contains vertex 15, in addition to its neighbors. Consider further that our method (see sub-section *Unsupervised community-based embedding*), with an appropriate parameter selection, will identify two user-centric communities after searching around vertex 11. The base community (*b*_11_), comprised of its neighbors, and another one (*e*_11_) that also incorporates vertex 12. This means that vertex 12 is characterized through its association with four vertices (including itself): *b*_5_, *b*_8_, *b*_12_ and *e*_11_. Association with *e*_11_ reinforces the assignment of label C to vertex *b*_12_.

**Table 2:** Vertex-centric communities lead to more personalized embeddings.

**A**																				
	*b*_0_	*b*_1_	*b*_2_	*b*_3_	*b*_4_	*b*_5_	*b*_6_	*b*_7_	*b*_8_	*b*_9_	*b*_10_	*b*_11_	*b*_12_	*b*_13_	*b*_14_	*b*_15_				
*v*_4_	0	1	0	0	1	1	1	1	0	0	0	0	0	0	0	0				
*v*_12_	0	0	0	0	0	1	0	0	1	0	0	0	1	0	0	0				
*v*_13_	0	0	0	1	0	0	1	0	0	0	0	1	0	1	1	0				
*v*_15_	0	0	1	0	0	0	0	0	0	0	0	0	0	0	1	1				
**B**																				
	*b*_0_	*b*_1_	*b*_2_	*b*_3_	*b*_4_	*b*_5_	*b*_6_	*b*_7_	*b*_8_	*b*_9_	*b*_10_	*b*_11_	*b*_12_	*b*_13_	*b*_14_	*b*_15_	*e*_1_	*e*_3_	*e*_11_	*e*_13_
*v*_4_	0	1	0	0	1	1	1	1	0	0	0	0	0	0	0	0	1	0	0	0
*v*_12_	0	0	0	0	0	1	0	0	1	0	0	0	1	0	0	0	0	0	1	0
*v*_13_	0	0	0	1	0	0	1	0	0	0	0	1	0	1	1	0	0	1	1	1
*v*_15_	0	0	1	0	0	0	0	0	0	0	0	0	0	0	1	1	1	1	0	1

**A**: Base Community and **B**: ARCTE.

### Related work

We now discuss the unique elements of the proposed user representation and classification framework compared to related works in *Relational classification*, *Local community detection* and *User profiling*.

#### Relational classification

The first related line of work refers to approaches in which the data do not inhabit a Euclidean space, but are described through their relations. We note that these methods are completely label-dependent. Our own approach is different in that the community-based social relation representation is done in a completely unsupervised way and may be reused in multiple experiments.

**Collective classification:** For classification on graphs, *collective classification* has been used extensively [13]. Such methods, however, have been outperformed by approaches using low-rank matrix representation. Specifically, LapEig used in a social network context [16] outperformed Link Based Classification (LBC) with relaxation labeling for collective classification [24] and the weighted vote relational neighbor classifier (wvRN) [25] with iterative classification [24, 26] on the ASU-Flickr dataset. ARCTE achieves even higher accuracy than LapEig on that dataset, so we elected not to include collective classification approaches in our study in order to leave room for more recent and competitive methods.

**Random walk similarity matrix methods:** Semi-supervised random walk methods for estimating vertex-to-vertex similarities also take into account the number of paths between vertices instead of the less distinctive measure of geodesic distance. Various *graph-kernels* or *similarity matrices* have been developed [12, 27]. However, they require the inversion of a matrix (*O*(*n*^3^)) as large as the input graph. Certain approaches [28] exploit advances towards near-linear system solutions [29] for diagonally dominant matrices or the small-world property of real-world graphs [30] to expedite the similarity matrix calculation, though they still require space quadratic to the number of samples. Finally, we note that the semi-supervised label diffusion method [28] has been outperformed by the low-rank matrix embedding approach—specifically by the RWModMax [17] method we included in our comparisons.

#### Local community detection

When a graph becomes too large, a sensible hypothesis is that one does not need access to the full graph structure in order to extract a community near a specific vertex. This concept has sparked the interest for local community detection methods, such as the random walk based Nibble algorithm [31] and its improvement PageRank-Nibble [32] that were used in constructing near-linear time spectral graph sparsifiers [33], solvers of symmetric, diagonally dominant systems [29] and conventional community detection [34]. The method by [32] relies on the calculation of a personalized, vertex-centric array of similarities for a seed vertex and a conductance *sweep* [31] of the sorted values to search for a good conductance cut.

We consider user-centric community detection as a step in our social relation latent representation algorithm that is distanced from the aforementioned approaches by the fact that we *a*) perform it in an unsupervised way for all users in a graph and it does not need to be seeded with a set of labeled users [34, 35], *b*) we use a novel heuristic for thresholding the similarity vector and capturing missing links instead of using costly sweeps, and *c*) we make computational improvements on the calculation of similarity vectors.

#### User profiling

Whereas we focus on topic-oriented user classification, other efforts attempt to use graph-based techniques to reveal other aspects of users’ profiles. For example, a study involved the identification of authoritative users in Twitter based on text content and links/interactions [6]. According to it, the latter type is more informative although feature fusion yielded the best results. Furthermore, graph-regularized non-negative matrix factorization of text features was successfully used to identify spam accounts in Twitter [36]. A PageRank variation was proposed by [37] to combat spam account link farms. Also noteworthy are methods that try to extract role similarity [38] and types [39] from structural complexity in user graphs.

Finally, it should be noted that some recent works have pointed to some limitations of graph-based approaches. In a recent study [40] a number of graph-theoretic features such as various centrality measures, counts and ratios of followers, friends, replies, etc. did not offer significant discrimination. Another study [41] leveraged user connectivity in the second of a two-step classification process. First, users were classified using text- and behavior-based features and then a neighbor voting scheme was applied for updating the labels, something that did not lead to improved accuracy. Yet, both of the aforementioned approaches did not fully leverage the potential of graph-based representations, since the first did not utilize user connectivity for calculating similarities, while the latter relied on neighbor voting which has been found to be inferior to low-rank graph representations [14].

## Proposed framework

We will now describe the means by which we depart from previous user classification approaches. Our proposed high-dimensional, community-based, binary representation is amenable to low-complexity, highly interpretable feature weighting techniques as in text classification. We extract a massive number of vertex-centric communities for the formation of a highly-redundant latent embedding (see sub-section *Unsupervised community-based embedding*) and then perform a supervised community weighting step (see sub-section *Supervised community weighting*) for adapting the embedding to the labeling. An overview of the proposed framework is depicted in Fig 1, while the computational process is formalized in Alg 2.

**Algorithm 2** Community-based user classification

 INPUT: *A*, *V*_*l*_, *V*_*u*_, *Y*_*l*_ and *dim*

 OUTPUT: *Y*_*u*_

  **Unsupervised part**

1: *X* = community_embedding(*A*, *dim*)      ▷ Community-based representation

  **Supervised part**

2: *X* = community_weighting(*X*)       ▷ Supervised representation adaptation

3: $h=\left\{ \begin{array}{l} \text{multi-label}\left(\text{supervised\_classifier}\left(X_{l},Y_{l}\right)\right)\\ \text{multi-label}\left(\text{semi-supervised\_classifier}\left(X_{l},X_{u},Y_{l}\right)\right) \end{array}\right.$   ▷ Hypothesis

  **Prediction**

4: *Y*_*u*_ = *h*(*X*_*u*_)                    ▷ Out-of-sample prediction

### Unsupervised community-based embedding

We now introduce ARCTE, an algorithm based on the extraction of vertex-centric communities for graph-based feature extraction. It produces a fine-grained representation of all vertices without being too computationally expensive or requiring huge amounts of storage space. We focus on two types of community with respect to each seed vertex: *a*) the set of base user-centric communities *C*_1_ = {*b*_*v*_}, where *b*_*v*_ is the base community around the seed vertex *v*, and *b*) the set of extended user-centric communities *C*_2_ = {*e*_*v*_}, where *e*_*v*_ is the extended community that results from identifying similar, but not directly connected vertices to the seed *v*. The final output is *X* = embed(*C*_1_ ∪ *C*_2_). As stated previously, each base community *b*_*v*_ = *N*(*v*) ∪ *v*, ∀*v* ∈ *V*. The merits of using such a community were described in sub-sub-section *Vertex-centric community embedding*. As for the second type of community, suppose that for any given seed vertex, we have a vector *k* of length |*V*| and that each element contains a value that encodes the similarity between user *v* and every user in the graph, including *v*. We can extract an extended user-centric community by appropriately truncating the similarity vector such that only users that exhibit high similarity (detailed in sub-sub-section *Fast similarity vector calculation*) to the seed are kept.

Truncating the similarity vector can be done in multiple ways, such as “sweeping” the sorted vector for a good conductance cut [31, 32]. Since we want to perform this truncation for all users, we opt for a faster approach. Given a similarity vector, sorted according to decreasing similarity, we select the fewest possible, highest ranking users, such that they comprise a strict superset of the corresponding base community. The sparsity of *k* (described in sub-sub-section *Fast similarity vector calculation*) guarantees a fast sorting process. Our motivation for this part is that if a non-adjacent vertex is a more probable random walk destination than the adjacent ones, then it is bound to be similar. We present a description in Alg 3. We denote by nnz(*k*) the non-zero element indices of vector *k*.

**Algorithm 3** ARCTE

INPUT: *A*, *ρ*_*eff*_, *ε*

OUTPUT: *X*

1: initialize *C*_1_, *C*_2_ ← ∅

2: **loop**[∀*u* ∈ *V*]

  **Step 1**—**Find base user-centric community**

3:  *b*_*u*_ ← *N*(*u*) ∪ *u*             ▷ Calculate base community

4:  *C*_1_ ← *C*_1_ ∪ *b*_*u*_          ▷ Add base community to the *C*_1_ set

  **Step 2—Find extended user-centric community**

5:  *k* ← get_similarity_vector(*u*, *ρ*_*eff*_, *ε*)   ▷ Reg. commute-times—Alg 4

6:  *k* ← sort(*k*)         ▷ Sort in decreasing order of similarity

7:  *e*_*u*_ ← ∅

8:  **loop**[*v* ∈ nnz(*k*)]      ▷ Go over all non-zero similarity vertices

9:   *e*_*u*_ ← *e*_*u*_ ∪ *v*               ▷ Start adding vertices

10:   **if** *b*_*u*_ ⊂ *e*_*u*_ **then**   ▷ If base community *strict* subset of extended …

11:    *C*_2_ ← *C*_2_ ∪ *e*_*u*_     ▷ … add extended community to the *C*_2_ set

12:    **break**

13:   **end if**

14:  **end loop**

15: **end loop**

16: *X* = embed(*C*_1_ ∪ *C*_2_)     ▷ Form community-based features—Eq 1

#### Fast similarity vector calculation

Similarity vectors for all seed users must be calculated and then sorted. To this end, working only on the non-zero elements is necessary for a scalable solution. Avoiding to propagate trivial values by assuming that similarities with distant vertices are almost zero leads to faster methods and storing only non-zero values results in sparse similarity vectors. In this section, we propose *an improved variation of fast, sparse, vertex-centric similarity vector calculation [32] by deriving a fast algorithm that approximates cumulative PageRank differences, which results in the discovery of more relevant vertices with fewer iterations*.

Denote by $k_{rw}^{\left(t\right)}\left(v\right)$ the probability of an agent randomly walking on a graph being on vertex *v* at time step *t* and by the row vector $k_{rw}^{\left(t\right)}$ the probability distribution for all vertices. The distribution after a random step is $k_{rw}^{\left(t+1\right)}=k_{rw}^{\left(t\right)}W$, where *W* = *D*^−1^ *A* is the Markov chain transition probability matrix and *D* is the diagonal degree matrix. By altering the random walk process to restart at each step to an initial distribution *s* with a given restart probability *ρ* ∈ [0, 1], $k_{rw}^{\left(t\right)}$ converges to a stationary distribution called the PageRank vector *k*_*pr*_. When *s* is anything but the uniform distribution, we call the resulting stationary distribution *personalized* PageRank [42]. We will consider *s* = *e*_*v*_, where *e*_*v*_ is a distribution with all probability concentrated on the seed position *v*, thus leading to *user-centric* similarity vectors. The user-centric PageRank is shown in Eq 2.

$$\begin{array}{c} k_{pr}^{\left(t+1\right)}=\rho e_{v}+\left(1-\rho \right)k_{pr}^{\left(t\right)}W, \end{array}$$ (2)

We base our approach on the concept of updating a single element *u* ∈ *V* per iteration, which has been adopted by different approaches [32, 43, 44]. The method described by [43] is for simultaneous calculation of multiple PageRank vectors by maintaining a heap, the relevant part of the work by [44] describes a method for calculating vertex-centric Katz score vectors also by maintaining a heap, whereas a lazy random walk based PageRank method is introduced by [32] where a queue replaces the heap, leading to lower complexity. A lazy random walk implies that there is a non-zero probability that the agent will perform a self-loop at any given time step *t*. We show in Eq 3 the single element update for a single PageRank vector by means of a non-lazy random walk.

$$\begin{array}{c} k_{pr}^{\left(t+1\right)}=k_{pr}^{\left(t\right)}+r^{\left(t\right)}I_{u}, \end{array}$$ (3)

where $r^{\left(t\right)}=\rho e_{v}-k_{pr}^{\left(t\right)}\left(I-\left(1-\rho \right)W\right)$ is the residual probability vector at time step *t* and *I*_*u*_ is a zero matrix with a single unit element at the *u*-th place of the main diagonal. In order to avoid propagating trivial values, only a significant element *u* from the residual is used per update; this means that only one transition probability row *W*_*u*:_ = *I*_*u*_ *W* is needed to update the residual. Eq 4 is the incremental rule for updating the residual.

$$\begin{array}{cc} r^{\left(t+1\right)} & =\rho e_{v}-\left(k_{pr}^{\left(t\right)}+r^{\left(t\right)}I_{u}\right)\left(I-\left(1-\rho \right)W\right)\\ & =r^{\left(t\right)}-r^{\left(t\right)}I_{u}+\left(1-\rho \right)r^{\left(t\right)}I_{u}W\\ & =r^{\left(t\right)}-r^{\left(t\right)}I_{u}+\left(1-\rho \right)r^{\left(t\right)}W_{u:}, \end{array}$$ (4)

One has to initialize the approximate solution as a zero vector $k_{pr}^{\left(0\right)}=\bar{0}$ and the residual $r_{pr}^{\left(0\right)}=e_{v}$ and update alternatively Eqs 3 and 4 until convergence. Since we multiplied *r*^(0)^ by ^1^/_*ρ*_, we need to also multiply the update in Eq 3 by *ρ* in order to calculate a probability distribution (i.e. summing to 1), if needed. However, we can avoid that by multiplying the resulting vector $k_{pr}^{\left(T\right)}$ by *ρ* after the final step, *T*. Following previous approaches [32, 44] we select an element *u* if it has a value ^*r*(*u*)^/_*d*(*u*)_ > *ε*, where *ε* is a predefined threshold.

The reason why it is preferable to use a non-lazy random walk over a lazy one in user-centric PageRank calculation is described in S2 Appendix.

For our second improvement, consider the vector *ck*_*δpr*_ of cumulative differences *k*_*δpr*_ between the PageRank distribution at step *t* in Eq 5.

$$\begin{array}{c} ck_{\delta pr}^{\left(t+1\right)}=ck_{\delta pr}^{\left(t\right)}+k_{\delta pr}^{\left(t+1\right)}=ck_{\delta pr}^{\left(t\right)}+k_{pr}^{t+1}-k_{pr}^{t}, \end{array}$$ (5)

The incremental rule for calculating *ck*_*δpr*_ is shown in Eq 6. We propose the alternative update of the rules in Eqs 6 and 4 for the calculation of cumulative PageRank differences. We initialize $k_{\delta pr}^{\left(1\right)}=k_{pr}^{\left(1\right)}-k_{pr}^{\left(0\right)}=e_{v}$. A derivation and further discussion can be found in S3 Appendix.

$$\begin{array}{c} ck_{\delta pr}^{\left(t+1\right)}=ck_{\delta pr}^{\left(t\right)}+\left(1-\rho \right)r^{\left(t-1\right)}W_{u}, \end{array}$$ (6)

The vector can be calculated as follows: one maintains a queue of all elements in the degree-normalized distribution *r* with probability exceeding the threshold *ε*. We perform *exactly one* iteration for each element (see S2 Appendix), by removing lazy steps since they result in some probability remaining in *r*. Any element in *r* that has now surpassed the threshold is appended to the queue. We repeat until $\frac{r(u)}{d(u)}<\varepsilon$, ∀*u* ∈ *V*. We formalize the above generalized technique in Alg 4. We denote by ./ the elementwise division operation and by *deg* the array of vertex degrees. The reason we perform this element-wise division is to get a similarity measure that is more focused on the locality of a target vertex to the seed than its high degree and is explained in S3 Appendix. One can substitute the Alg 5 for the lazy random walk PageRank [32]. In order to utilize our improved versions without a laziness factor one must substitute Algs 6 and 7 for PageRank and cumulative PageRank differences respectively. We also denote by *w*_*vu*_ the transition probability from vertex *v* to *u*.

**Algorithm 4** Vertex-centric Similarity Vector Calculation

INPUT: *W*, *v*_*seed*_, *ρ*, *ε*

OUTPUT: *k*^(*t*+1)^, *r*^(*t*+1)^

1: initialize *k*^(0)^ ← 0, $k^{\left(0\right)}\left(v_{seed}\right)\leftarrow \{\begin{array}{c} 0\;\text{if}\;\text{PageRank}\\ 1\;\text{if}\;\text{Cumulative}\;\text{PageRank}\;\text{differences} \end{array}$

2: initialize $r^{\left(0\right)}\leftarrow \bar{0}$, *r*^(0)^(*v*_*seed*_)←1

3: **loop**[while $\exists V_{r}\subseteq V:\forall v\in V_{r},\frac{r^{\left(t\right)}\left(v\right)}{d(v)}\geq \varepsilon$]

4:  **loop**[∀*v* ∈ *V*_*r*_]

5:   *k*^(*t*+1)^, *r*^(*t*+1)^ ← update(*k*^(*t*)^, *r*^(*t*)^, *v*, *ρ*)            ▷ Algs 5, 6 or 7

6:  **end loop**

7: **end loop**

8: *k*^(*t*+1)^ ← *k*^(*t*+1)^./*d*           ▷ Calculate regularized commute-times

**Algorithm 5** PageRank Lazy Update

INPUT: *W*, $k_{\lambda pr}^{\left(t\right)}$, *r*^(*t*)^, *v*, *ρ*, *λ*

OUTPUT: $k_{\lambda pr}^{\left(t+1\right)}$, *r*^(*t*+1)^

1: **loop**[while $\frac{r^{\left(t\right)}}{d(v)}\geq \varepsilon$]

2:  $k_{\lambda pr}^{\left(t+1\right)}\left(v\right)\leftarrow k_{\lambda pr}^{\left(t\right)}\left(v\right)+\rho r^{\left(t\right)}$

3:  **loop**[∀*u* ∈ *N*(*v*)]

4:   *r*^(*t*+1)^(*u*)←*r*^(*t*)^(*u*) + (1 − *ρ*)(1 − *λ*)*r*^(*t*)^(*v*)*w*_*vu*_

5:  **end loop**

6:  *r*^(*t*+1)^ ← (1 − *ρ*)*λr*^(*t*)^

7: **end loop**

**Algorithm 6** PageRank Limit Update

INPUT: *W*, $k_{pr}^{\left(t\right)}$, *r*^(*t*)^, *v*, *ρ*_*eff*_

OUTPUT: $k_{pr}^{\left(t+1\right)}$, *r*^(*t*+1)^

1: $k_{pr}^{\left(t+1\right)}\left(v\right)\leftarrow k_{pr}^{\left(t\right)}\left(v\right)+\rho_{eff}r^{\left(t\right)}\left(v\right)$

2: **loop**[∀*u* ∈ *N*(*v*)]

3:  *r*^(*t*+1)^(*u*)←*r*^(*t*)^(*u*) + (1 − *ρ*_*eff*_)*r*^(*t*)^(*v*)*w*_*vu*_

4: **end loop**

5: *r*^(*t*+1)^(*v*)←0

**Algorithm 7** Cumulative PageRank Differences Update

INPUT: *W*, $ck_{\delta pr}^{\left(t\right)}$, *r*^(*t*)^, *v*, *ρ*_*eff*_

OUTPUT: $ck_{\delta pr}^{\left(t+1\right)}$, *r*^(*t*+1)^

1: **loop**[∀*u* ∈ *N*(*v*)]

2:  $ck_{\delta pr}^{\left(t+1\right)}\left(u\right)\leftarrow ck_{\delta pr}^{\left(t\right)}\left(u\right)+\left(1-\rho_{eff}\right)r^{\left(t\right)}\left(v\right)w_{vu}$

3:  *r*^(*t*+1)^(*u*)←*r*^(*t*)^(*u*) + (1 − *ρ*_*eff*_)*r*^(*t*)^(*v*)*w*_*vu*_

4: **end loop**

5: *r*^(*t*+1)^(*v*)←0

**Fig 3:**
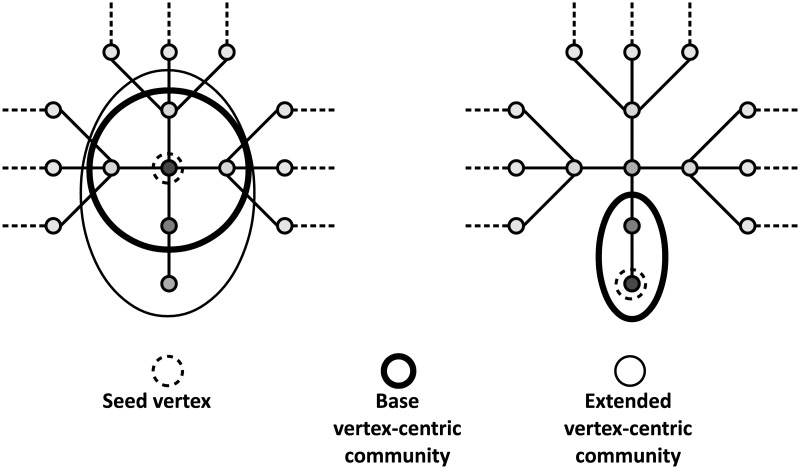
Vertex-centric communities on degree-1 vertices. We do not calculate extended vertex-centric communities for seed vertices of degree equal to 1. However, they may be included in extended communities centered on other vertices.

Finally, we perform a local search only for seed vertices with degree above 1. Given that the similarity vector *k* is an approximation of a *degree-normalized* random walk with restart, it is impossible for any vertex to have a larger similarity score than thxze unit degree seed or the adjacent. Naturally, we extract only the base community for such seed vertices, although their representation can also be improved with ARCTE. As shown in Fig 3, these one-degree vertices may still participate in the extended user-centric community of some other vertex, as the adjacent vertices may be penalized due to high degree.

#### Threshold parameter selection

The threshold parameter *ϵ* regulates the approximation to the true PageRank vector. No formal methodology has been proposed regarding its selection in vertex-centric methods. In a recent empirical discussion [34] the authors claim that *ϵ* ∈ [10^−5^, 10^−6^] offers good local exploration without increasing the computational complexity prohibitively. We frame this rule by further observations of our own. We denote by *d*_*ave*_(*v*), *d*_*max*_(*v*), *d*_*min*_(*v*) the average, maximum and minimum degrees of the neighborhood *N*(*v*). For example, we give the definition of the maximum neighborhood degree: *d*_*max*_(*N*(*v*)) = max(*d*(*u*)), ∀*u* ∈ *N*(*v*).

a) We calculate an effective threshold value *ϵ*_*eff*_ that takes into account the one-hop structure around each seed vertex *v*_*seed*_. Specifically, we calculate $\epsilon_{eff}=\epsilon \frac{\log(1+d\left(v_{seed}\right))}{\log(1+d_{ave}\left(v\right))}$ to encourage a more strict approximation in case the degrees of the adjacent vertices are relatively larger than the degree of the seed. This accommodates for the greater complexity in the local graph structure.
b) There is an implicit, hard upper bound *ϵ*_*max*_. Consider the residual distribution *r* after one iteration. An amount of probability *averaging* to ^(1 − *ρ*)^/_*d*(*v*_*seed*_)_ will be on the adjacent vertex positions in *r*. At least one such value must be higher than the corresponding vertex’s degree normalized threshold ^*ϵ_eff_*^/_*d*_(*vadj*)__ in order for a second iteration to take place. As such, *ϵ*_*max*_ = ^*d*_*max*_(*v*)^/_*d*(*v*_*seed*_)_.
c) We also apply a soft lower bound. Since we are interested in local graph exploration, we do not want to overly spend computational resources by propagating values needlessly further away from each seed vertex. As such, we penalize the *ϵ*_*eff*_ if it is larger than the value that guarantees a probability push on *all* adjacent to the seed vertices. Specifically, we calculate *ϵ*_*eff*_ = ^*ϵ*_*eff*_+*d*_*min*_(*v*)^/_2_

#### Parallelization

**Fig 4:**
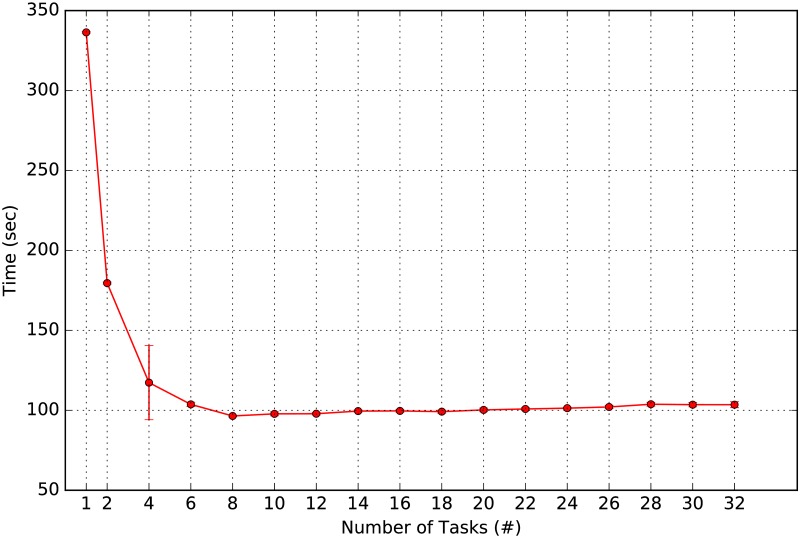
Parallelized feature extraction time. ARCTE execution time versus number of parallel tasks on ASU-Flickr dataset.

We note that each user-centric similarity vector and subsequent community detection is *independently calculated*. Furthermore, the transition probability matrix *W* is read-only for the purpose of ARCTE and does not require the acquisition of a lock in order to read from it. Under a *shared physical memory* model, ARCTE is an *embarassingly parallel* algorithm, which means that each vertex seed may be mapped to a separate processor (such exploitation of independent processes in graphs for parallelism has been successfully used before, e.g., in the calculation of shortest paths [45]). The reduction of the parallel outputs to a single feature matrix is identical to the serial result. For instance, if the machine used for the experiments had 8 cores we would theoretically expect a speed-up of *up to* 8x for 8 parallel tasks (threads, processes), which is never observed in practice given the overhead required to prepare and initiate the tasks as well as to reduce the results into a single matrix. Fig 4 depicts the execution time versus number of tasks. Given the fact that multiprocessors are capable of handling an additional small number of asynchronous tasks, we observe that there is no further noticeable degradation of performance as one creates tasks beyond 8.

We adopt the following simple parallelization scheme. Let *t*_*num*_ be the number of tasks we want to initiate. We sort the degree array in increasing order. We then map to the task *t* the vertices corresponding to the degree values found in the sorted degree array positions *t* + *i* × *t*_*num*_ where *i* ∈ [0, ^|*V*|^/_*t*_*num*__ − 1]. This way, vertices are distributed in a degree-wise balanced way among tasks.

### Supervised community weighting

In the previous sub-section we described an unsupervised method for extracting user-centric communities for embedding users in a latent space. Depending on the annotation, some of the vertices participating in these communities might be very important predictors, whereas others may be completely uncorrelated to all labels. We introduce a community weighting step for boosting the importance of communities in the latent representation, before learning a hypothesis for multilabel user classification.

The features extracted by ARCTE are high-dimensional, extremely sparse binary representations. Therefore, we base our approach on term weighting methods from the text classification literature [46], where binary representations are commonly used. We multiply each feature *j* with a weighting value *w*_*j*_ that encodes the significance of the corresponding community. The value *w*_*j*_ is calculated such that it addresses the following natural observations:

- Large communities imply weaker vertex intra-community participation.
- Communities with multiple labeled vertices are probably potent predictors.

We address the first point via *inverse vertex frequency* weighting [23] and the second by calculating the dependence between features and labels via the *χ*^2^ statistical test. For every feature *j* we calculate *w*_*j*_ = *ivf*(*j*) × *χ*^2^(*j*).

The first term is calculated as follows: We divide all non-zero elements of column *X*_:*j*_ by a function of the number of vertices in the corresponding community *c*_*j*_, i.e. *X*_:*j*_ ← ^*X*_:*j*_^/_*f*(|*c_j_*|)_. After empirical experiments we conclude that a good choice for *f*(.) is the square root of the logarithm, although other functions (e.g., the logarithm) also produce adequate results.

For the second term, we first form a *contingency matrix* *M*, that holds a statistical dependence score for all feature/label (*dim* × *l*) combinations. We use the *χ*^2^ test over alternatives such as mutual information and information gain, due to its simplicity and success in text-based feature selection [47] and weighting [46]. We calculate each *M*_*jl*_ as in Eq 7:

$$\begin{array}{c} M_{jl}=\chi_{j,l}^{2}=\frac{{|V|\left(A\cdot E-D\cdot B\right)}^{2}}{\left(A+D)(B+E)(A+B)(D+E\right)}, \end{array}$$ (7)

where *A* is the number of co-occurences between *j* and *l*, B the number of times *j* occurs without *l*, *D* is the number of times *l* occurs without *j* and *E* the number of times neither *j* nor *l* occur. Aggregating the scores across labels to extract one value per feature can be done in multiple ways e.g., by getting the maximum or the average value. We opted for a more principled method by using the *peak signal-to-noise ratio*. Each aggregated *χ*^2^(*j*) value is calculated as in Eq 8, normalized by within-label variability (wlv) as in Eq 9:

$$\chi_{j}^{2}=\frac{\text{max}\left(\chi_{j,l}^{2}\right)-\text{min}\left(\chi_{j,l}^{2}\right)}{\text{wlv}\left(M\right)}$$ (8)

$$\text{wlv}\left(M\right)=\sqrt{\frac{1}{\left|L\right|}\underset{l\in L}{\sum }\sigma^{2}\left(M_{:l}\right)},$$ (9)

We further pass the *χ*^2^ term through a logarithmic function ($\log(1+\chi_{j}^{2})$), in order to avoid imbalanced boosting weights. The community weighting process is summarized in Alg 8. We note that we store the binary feature matrix *X* and the community weight vector *w* separately in order to keep the unsupervised and supervised parts of the user classification framework separated. We only apply the weighting before training the classifier.

**Algorithm 8** Community weighting

INPUT: *X*

OUTPUT: *X*_*w*_

 **Step 1**: *ivf* term calculation

1: **loop**[∀*c*_*j*_ ∈ *C*]

2:  $ivf\left(j\right)\leftarrow \frac{1}{\sqrt{\log(|c_{j}|)}}$

3: **end loop**

 **Step 2**: *χ*^2^ term calculation

4: **loop**[∀*c*_*j*_ ∈ *C*]

5:  **loop**[∀*l* ∈ *L*]

6:   $M_{jl}\leftarrow \chi_{jl}^{2}$             ▷ Contingency matrix—Eq 7

7:  **end loop**

8: **end loop**

9: **loop**[∀*c*_*j*_ ∈ *C*]

10:  *χ*^2^(*j*)←PSNR(*M*_*j*:_)      ▷ Peak signal-to-noise ratio—Eqs 8 and 9

11: **end loop**

  **Step 3**: Community weighting

12: **loop**[∀*c*_*j*_ ∈ *C*]

13:  *X*_:*j*, *w*_ ← *X*_:*j*_ × *ivf*(*j*) × log(1+*χ*^2^(*j*))

14: **end loop**

As a final note, we also mention that there exist related supervised methods (see recent survey [48]), such as wrappers that search for a good feature *subset*. These methods employ heuristic searches in the feature power-set space and are thus computationally expensive. We followed a simpler approach, as the matrix *M* is calculated in *O*(|*L*||*C*|). Of course, substituting our own community weighting step with such a method could be a possible extension.

## Evaluation

In this section we describe the annotated datasets, evaluation measures, competing methods and experimental setup.

### Datasets

For our comparative study, we introduce a new dataset for graph-based classification, called *SNOW 2014 Graph* (https://github.com/MKLab-ITI/reveal-graph-embedding). We also utilize datasets accessed from the Arizona State University (ASU) repository (http://socialcomputing.asu.edu) and the Insight Resources (IR) repository (http://mlg.ucd.ie/index.html#data). It should be noted that user connections in the relevant OSNs are directed, whereas the ASU graphs provided are undirected and as such some kind of edge post-processing is assumed to have been applied. Table 3 presents some basic statistics for these datasets.

**Table 3:** Basic graph dataset statistics. We denote by *d*_*max*_, *d*_*ave*_ and *l*_*ave*_ the maximum degree, average degree and average number of labels per user respectively.

Datasets	Vertices	Edges	Labels	d_max_	d_ave_	Labeled Users (#)	l_ave_
SNOW2014G	533,874	949,661	90	16,287	4	10,992	2.53
ASU-Flickr	80,513	5,899,882	195	5,706	146	80,513	1.4
ASU-YouTube	1,134,890	2,987,624	47	28,754	5	31,684	1.6
IRMV-PoliticsUK	419	11,349	5	317	110	419	1

#### SNOW 2014 Graph (SNOW2014G)

**Fig 5:**
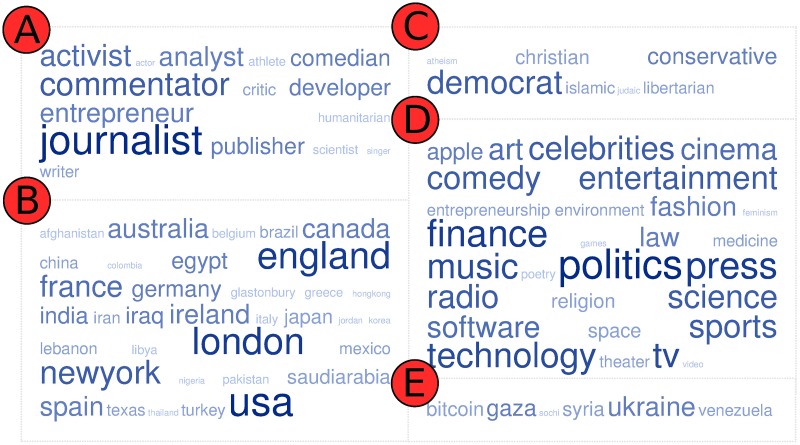
User labels in SNOW2014G. Starting from upper-left word cloud and going clockwise: A: Attributes (e.g., occupation). B: Geographical association. C: Religious or political stance. D: Associated themes. E: News story relevance.

We extracted mention and retweet social interactions to form the graph edges from the tweet collection introduced in the SNOW 2014 Data Challenge [49]. The labels we gathered belong to various types of user attribute, as depicted in Fig 5. The procedure for extracting and annotating the graph is described in S1 Appendix. We opted for a *connected* and *undirected* graph of users *in order to make the method comparisons fair*. We required the former quality since *disconnected graphs are problematic for the application of spectral methods* (see ASU-YouTube) and the latter because *not all competing methods are applicable to directed graphs*.

#### ASU-Flickr (ASU-FR) [14]

Graph vertices represent users in the Flickr (https://www.flickr.com/) image and video hosting platform. Flickr users may follow each other and also subscribe to specific interest groups.

#### ASU-YouTube (ASU-YT) [50]

Graph vertices represent users in the YouTube (https://www.youtube.com/) video sharing website. Apart from uploading videos, users form a subscription graph among them and also subscribe to various interest groups. It was introduced in [50] and has been used to evaluate scalable algorithms [20] by keeping the labels with more than 500 vertices as ground truth. Since the graph used in [20] was disconnected, we perform one more post-processing step and keep only the largest connected component, since the spectral methods in our comparative study *could not converge* for a disconnected graph even after 4 days of continuous execution.

#### Insight Resource Multiview (IRMV) [51]

These are five multi-view datasets with manual annotation of user stances (e.g., political or sports). In order to perform similar experiments as with our own SNOW2014G dataset, we extracted an undirected graph that integrates two social interaction graph views, namely a mention and a retweet graph (see S1 Appendix). Since they are all small and of similar size, we will report only on results from the IRMV-PoliticsUK dataset.

### Measures

Since we are dealing with multi-label classification, we report micro- and macro-averages of the F1 measure. We count for each label *l* ∈ *L* the number of true positives (*tp*_*l*_), false positives (*fp*_*l*_) and false negatives (*fn*_*l*_). If the denominator in F1 is zero for a label in the case of macro-averaging, we consider it undefined but we equal it to zero in order to get a numerical average. The F1 micro- and macro-averages are defined in Eqs 10 and 11 respectively.

$$\begin{array}{cc} F1_{micro} & =\frac{2\sum_{l\in L}tp_{l}}{2\sum_{l\in L}tp_{l}+\sum_{l\in L}fp_{l}+\sum_{l\in L}fn_{l}} \end{array}$$ (10)

$$\begin{array}{cc} F1_{macro} & =\frac{1}{\left|L\right|}\sum_{l\in L}\frac{2tp_{l}}{2tp_{l}+fp_{l}+fn_{l}}, \end{array}$$ (11)

### Competing methods

We present a short description of the competing methods. Wherever the implementation source is not stated, we used our own Python implementations.

#### Laplacian Eigenmaps (LapEig) [21]

This technique has been used for embedding OSNs [16]. We calculate the *dim* eigenvectors corresponding to the *dim* smallest eigenvalues of the symmetric normalized Laplacian $D^{-{}^{1}\;{/}_{2}}LD^{-{}^{1}\;{/}_{2}}$, where *L* = *D* − *A* is the Laplacian; excluding the one corresponding to the zero-valued eigenvalue.

#### Replicator Eigenmaps (RepEig) [52]

This is the name we give to the computation of the eigenmaps of the Replicator matrix *R* = *λ*_*max*_ *I* − *A*, where *λ*_*max*_ is the largest eigenvalue of *A*. While the Laplacian is related to probability preserving random walks, the Replicator describes a diffusion process of an agent that transitions simultaneously to all adjacent vertices.

#### Random Walk Modularity Maximization (RWModMax) [17]

Circumventing the modularity measure’s resolution limit [53], the random walk modularity measure assesses statistical significance of communities based on random walk paths instead of edges. The top *dim* eigenvectors of the random walk modularity matrix are computed. We used the implementation provided by the authors (https://github.com/rdevooght/RWModMax).

#### Deepwalk [18]

This method treats random walk paths as documents and then applies deep representation learning to embed the users in a low-dimensional space. We used the implementation provided by the authors (https://github.com/phanein/deepwalk).

#### LINE [19]

This is an efficient method that attempts to preserve both the first and the second order connectivity of the vertices in a low-rank matrix embedding. We used the implementation provided by the authors (https://github.com/tangjianpku/LINE).

#### Louvain [54]

A hierarchical disjoint community detection method considered state-of-the-art in terms of both speed and community quality [22]. We used a Python implementation available online (https://bitbucket.org/taynaud/python-louvain).

#### Edge Clustering (EdgeCluster) [20]

This is an overlapping community detection method, specifically designed for extracting features from OSN graphs. It is an edge-centric k-means variant that exploits the adjacency matrix sparsity to calculate only relevant similarities. We used the implementation provided by the authors (http://leitang.net/social_dimension.html).

#### Multiple Resolution Overlapping Communities (MROC) [23]

A hierarchical community detection algorithm also designed for OSN graphs. It iteratively merges communities from highest to lowest resolution based on their similarity. In order to avoid the calculation of all possible pairs, the merging is based on heuristics such that a binary tree community hierarchy is produced.

#### Cluster Affiliation Model For Big Networks (BigClam) [55]

A fast, overlapping community detection method based on a generative matrix factorization model. We used the implementation from the Stanford Network Analysis Project (SNAP) website (http://snap.stanford.edu/).

#### Order Statistics Local Optimization Method (OSLOM) [56]

A hierarchical, overlapping community detection method. A number of clean-ups can be ran for each level to better assess community significance, although this increases runtime. We used the authors’ reference implementation (http://www.oslom.org/).

#### Base Communities (BaseComm)

These are defined as: *X* = *A*_*sup*_ + *I*, where *A*_*sup*_ is the binary support matrix of *A*.

For OSLOM, Louvain and MROC we used the hierarchy of communities for the formation of *X*. For the eigenvector calculations performed in the spectral methods LapEig and RepEig we used the ARPACK [57] package implementation of the Implicitly Restarted Lanczos method. For sparse matrices, the method complexity may reach *O*(*i*|*E*|), otherwise it scales as *O*(*i*|*V*|^2^), where *i* is the number of iterations. Finally, some discussion on further alternative competing methods and the reasons for not including them in our study were discussed in the related work sub-sub-section *Relational classification*.

### Supervised learning

We opted for the use of the LIBLINEAR [58] linear Support Vector Machine classifier due to its linear complexity. We use it as the base of a One-vs-All multi-label scheme to produce a ranking of labels for each vertex. We opted for One-vs-All for the experiments in this study due to its low execution time, since multiple methods and parameter combinations were tested. Of course, there are other, more elaborate alternatives [15]. Following [14, 17], we assume the true number of labels for each vertex to be known. Our validation framework is as follows: we split the dataset into a training *V*_*l*_ and a testing *V*_*u*_ set via random sampling, ascertaining there is at least one training sample for each label in each set. We report performance measures across multiple training sample percentages. We perform 10 trials for each percentage in order to assess with good confidence the reported F-measure. The data-splits in these trials are shared across the competing methods in order to ensure a fair comparison.

### Parameter selection

All experiments were performed on an Intel^©^ Core^™^i7-4770K, with 8 cores at 3.50GHz and 15.6 GiB main memory. The parameters *θ* per algorithm are summarized in Table 4. An x-mark (✘) symbol means that the method failed to extract features even after days of execution. Wherever available, we used the parameters proposed in the respective studies [16–20, 23]. For Deepwalk, *γ* is the number of sampled walks, *w* the window size and *λ* the latent dimension number and we used the parameters proposed in the original paper [18]. For LINE, *dim* is the dimensionality of the embedding, *ρ*_0_ is the learning rate, *K* is the number of negative samples and *T* the number of mini-batches. As for the dimensionality, the value we report refers to the LINE embedding based on one order graph similarity. Following the original article, we use the first and second order version and as such the total dimensionality is twice the number we report. Regarding OSLOM, *r* and *hr* refer to the number of clean-up runs for the lowest and the higher hierarchical levels respectively. We selected as high values as possible, being constrained by the increase in runtime. As for BigClam, we tried different numbers of clusters in the range [10, 10000] and we kept the ones that led to the best results. For MROC, a parameter defines the maximum community size for which merging is allowed; we set *α* = 1000 as advised by the authors. As for RepEig and LapEig, we selected the best dimensionality *dim* ∈ {50, 100, 200, 300, 500, 1000} where possible. We considered the SVM hardness *C* as an additional parameter and we tried the following values: *C* ∈ {1, 5, 10, 50, 100, 200, 500, 1000}. Greater *C* values *significantly slow down model fitting*. We found that for the majority of methods, *C* = 1 was the choice that yielded the best performance, balanced between Macro and Micro F1. Our observation was that specifically for the spectral methods (i.e. LapEig, RepEig and RWModMax) an increase in *C* brought consistent improvement. Furthermore, for the low-rank representation approaches we found that it is best not to fit the intercept parameter since the features were centered. Conversely, for community-based embeddings we did fit the intercept after normalizing each row to 1. Parameter selection for ARCTE is described in the next section.

**Table 4:** Method parameters.

Methods	*θ*	SNOW2014G	ASU-FR	ASU-YT	IRMV-PoliticsUK
ARCTE	*ρ*, *ε*, *C*	0.1, 10^−5^, 1	0.1, 10^−5^, 1	0.1, 10^−5^, 1	0.1, 10^−5^, 200
LapEig	*dim*, *C*	1000, 10	500, 50	500, 1000	50, 10 [16]
RepEig	*dim*, *C*	500, 10	1000, 50	500, 100	50, 50
RWModMax	*dim*, *C*	✘	1000, 500 [17]	✘	50, 10
Deepwalk	*γ*, *w*, *λ*, *C*	80, 10, 120, 1	80, 10, 120, 1 [18]	80, 10, 120, 1 [18]	80, 10, 120, 1
LINE	*dim*, *ρ*_0_, *K*, *T*, *C*	128, 0.025, 5, 10, 1	128, 0.025, 5, 10, 1	128, 0.025, 5, 10, 1 [19]	128, 0.025, 5, 10, 1
EdgeCluster	*dim*, *C*	5000, 1	10000, 1 [20]	1000, 1 [20]	200, 5
MROC	*α*, *C*	1000, 1	1000, 1 [23]	1000, 1 [23]	1000, 200
Louvain	*C*	1	1	1	100
BigClam	*dim*, *C*	1000, 1	50, 1	500, 1	50, 1
OSLOM	*r*, *hr*, *C*	10, 10, 1	10, 10, 1	10, 10, 1	50, 50, 1
BaseComm	*C*	1	1	1	100

## Results

### Similarity vector comparison

#### Similarity vector calculation for user classification

**Fig 6:**
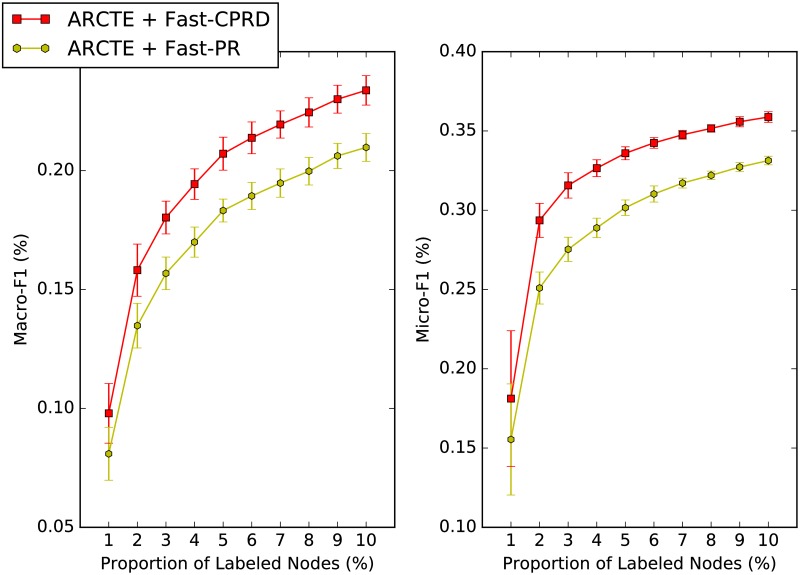
Similarity vector method comparison for user classification on SNOW2014G.

We compare the performance of our fast Cumulative PageRank differences (Fast-CPRD) method with the fast PageRank (Fast-PR) method, which we derive by substituting the similarity vector calculation step with Algs 7 and 6 respectively. Fig 6 depicts the results for the SNOW2014G dataset and we use for both methods the parameters from Table 4. We note that the Fast-CPRD method pushes probability values to the positions of the similarity vector *k* that correspond to the neighbors of the seed vertex from the *first* iteration. This guarantees that the neighbors will have non-zero values when the truncation operation is attempted. In our ARCTE variation that utilizes the Fast-PR method, we only try to extract an extended vertex-centric community around a seed vertex only when its neighbors have non-zero values; otherwise only the base vertex-centric community is extracted.

#### Similarity vector calculation efficiency

We also report the efficiency of vertex-centric similarity vector calculations. We made comparisons for the ARCTE parameters given in Table 4 for the three datasets. In the case of PageRank calculation with a restart probability *ρ*_*eff*_ and a laziness factor, we assume *λ* = ^1^/_2_, which implies *ρ* = ^*ρ*_*eff*_(1 − *λ*)^/_1 − *ρ*_*eff*_*λ*_. We do this so that both the Lazy-PR [32] and the Fast-PR method calculate an approximation to the same PageRank vector. We notice both a significant speed-up in execution time and a smaller number of operations for the PageRank comparison. Furthermore, we also compare the times with our Cumulative PageRank differences adaptation, which requires the exact same number of limit push operations as the fast PageRank method. We expect it to be marginally slower than the fast PageRank because the Cumulative PageRank differences push operation is a little heavier in computations. Evidently, the slightly slower execution of Fast-CPRD is justified by its increased predictive performance over Fast-PR (see previous paragraph). The execution times reported are averaged across 5 runs for each dataset and the measurements were performed using the Python profiler (https://docs.python.org/3.4/library/profile.html). The results are given in Table 5. The speed-up reported is with respect to the Lazy-PR method [32].

**Table 5:** Similarity vector calculation speed-up.

			SNOW2014G	ASU-FR	ASU-YT	IRMV-PoliticsUK
Lazy-PR	Total	# op	608,001,748	22,258,525	1,773,052,180	1,139,681
		*T* sec	1,381.92	74.37	4,125.85	3.42
	Per Node	# op	1,138.85	276.46	1,562.31	2,766.22
		*T* sec	2.59 ⋅10^−3^	0.92 ⋅10^−3^	3.64 ⋅10^−3^	8.31 ⋅10^−3^
Fast-PR	Total	# op	387,201,722	11,636,097	1,153,653,890	619,010
		*T* sec	817.91	43.02	2,476.17	1.73
	Per Node	# op	725.27	144.52	1,016.53	1,502.45
		*T* sec	1.53 ⋅10^−3^	0.53 ⋅10^−3^	2.18 ⋅10^−3^	4.19 ⋅10^−3^
	Speed-up	# op	36.32%	47.72%	34.94%	45.69%
		*T* sec	40.81%	42.15%	39.98%	49.61%
Fast-CPRD	Total	# op	387,201,722	11,636,097	1,153,653,890	619,010
		*T* sec	820.09	48.06	2,590.37	1.92
	Per Node	# op	725.27	144.52	1,016.53	1,502.45
		*T* sec	1.54 ⋅10^−3^	0.6 ⋅10^−3^	2.28 ⋅10^−3^	4.66 ⋅10^−3^
	Speed-up	# op	36.32%	47.72%	34.94%	45.69%
		*T* sec	40.66%	35.38%	37.22%	43.91%

### Parameter perturbation

#### ARCTE parameter selection

**Fig 7:**
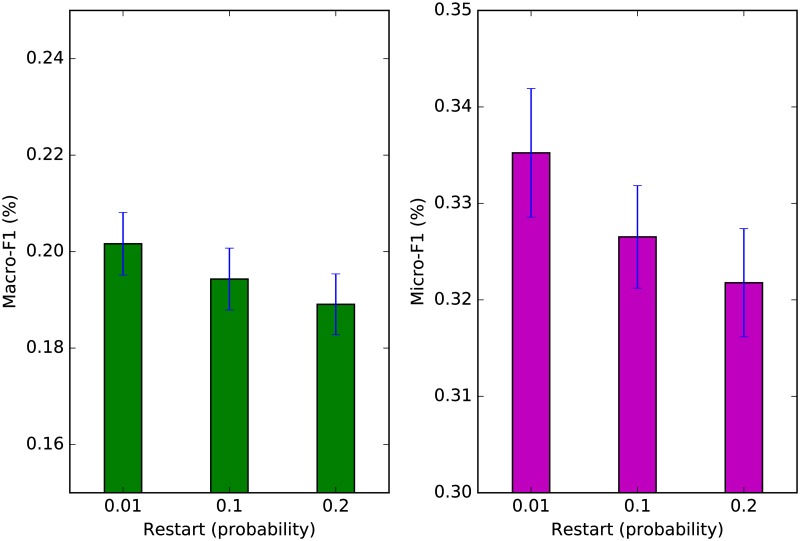
ARCTE performance on SNOW2014G: restart probability perturbation.

**Fig 8:**
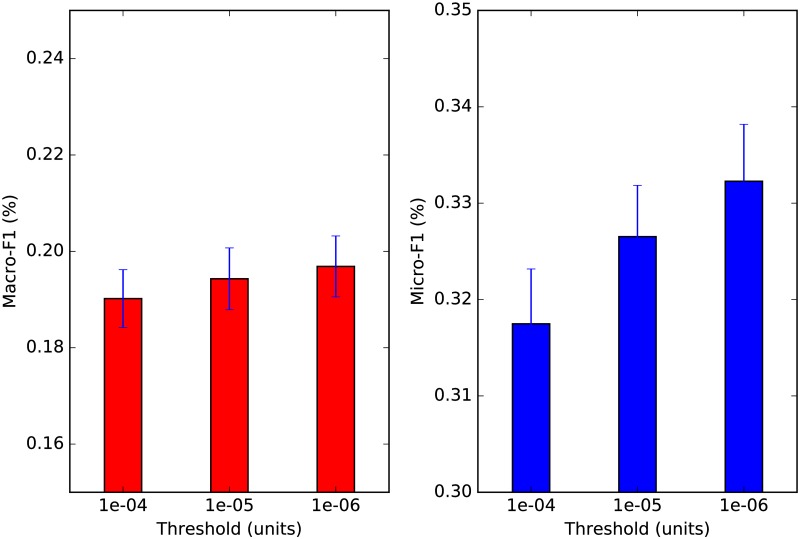
ARCTE performance on SNOW2014G: approximation threshold perturbation.

The calculation of our vertex-centric similarity vector is dependent on two variables: *a*) the restart probability *ρ*, and *b*) the threshold *ϵ*. A smaller restart probability encourages exploration such that vertices further from the seed have the chance to be ranked higher. As *ρ* → 0, the random walker performs a random walk without restart. A smaller threshold parameter leads to a better approximation to the true similarity vector as defined in Eq 2. Fig 7 depicts the performance of ARCTE for *ϵ* = 10^−5^ and variable *ρ* and Fig 8 depicts the performance of ARCTE for *ρ* = 0.1 and variable *ϵ* for a 4% training set in the SNOW2014G dataset. As a general rule, smaller *ρ* and *ϵ* values lead to better results. The only caveat here is that this also leads to larger execution times. Specifically, for our implementation, we note a 26.61% decrease in feature extraction time for ARCTE with *ρ* = 0.1, *ϵ* = 10^−4^ and a 30.83% increase for *ρ* = 0.1, *ϵ* = 10^−6^ compared to the parameters we report in Table 4. Similarly, we get a 39.37% decrease for *ρ* = 0.2, *ϵ* = 10^−5^ and a 183.06% increase for *ρ* = 0.01, *ϵ* = 10^−5^. In order to balance a performance showcasing the strength of ARCTE and manageable execution times, we opted for more moderate parameter values (*ρ* = 0.1, *ϵ* = 10^−5^) for the series of comparative experiments in sub-sub-section *User classification performance*.

#### Classifier parameter perturbation

**Fig 9:**
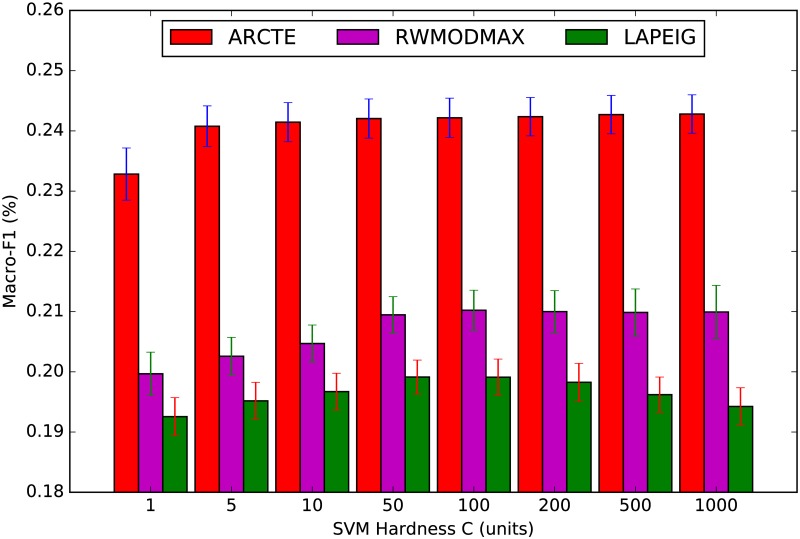
SVM C parameter perturbation on ASU-Flickr.

The SVM hardness *C* is also an important parameter in this series of experiments, as shown also in the RWModMax paper [17]. We show the effects of varying this parameter in Fig 9 for the ASU-Flickr dataset. On the x-axis, we perturb the linear SVM C parameter and we show the Macro-F1 measure on the y-axis. The results are similar for all training sets, but for reasons of space consumption we elected to report results only for the 4% training set. Generally, the perturbation of *C* does not lead to extreme variations, although we see that ARCTE is somewhat less dependent on this parameter when compared with the most competitive low-rank representation methods for the ASU-Flickr dataset.

### Feature weighting impact

**Fig 10:**
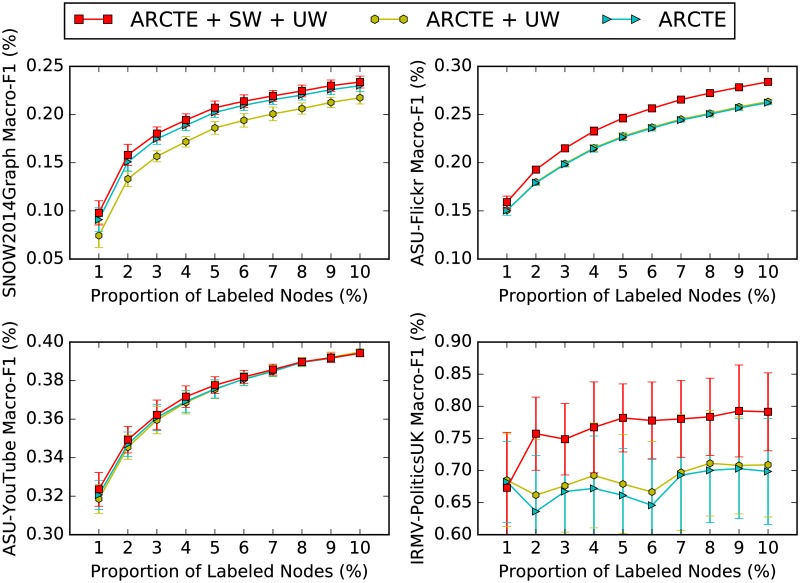
Community weighting impact.

We show the impact of the community weighting method we introduced in sub-section *Supervised community weighting*. We report ARCTE F1-Macro in Fig 10. By the label “ARCTE” we denote the performance based on features without any weighting. By “ARCTE + UW” we denote ivf community normalization and by “ARCTE + SW + UW” we denote both supervised and unsupervised weighting as described in sub-section *Supervised community weighting*. We see that in all cases, the supervised community weighting step improves the F-score performance consistently and is a clear improvement compared to simple unsupervised weighting [23].

### Comparison with existing methods

#### User classification performance

**Fig 11:**
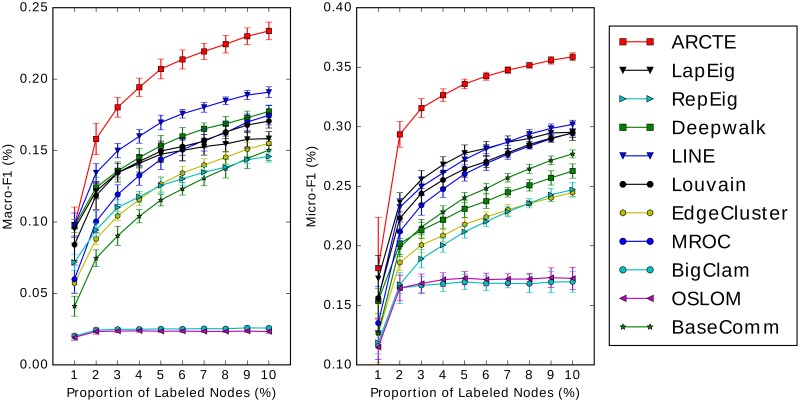
Performance on SNOW2014G (best viewed in color).

**Fig 12:**
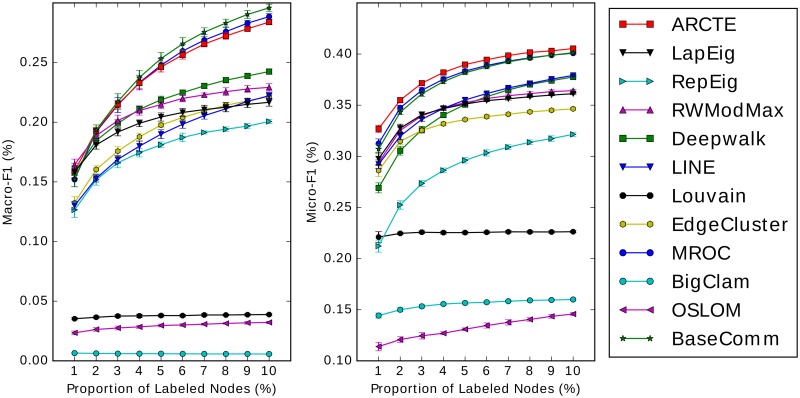
Performance on ASU-Flickr (best viewed in color).

**Fig 13:**
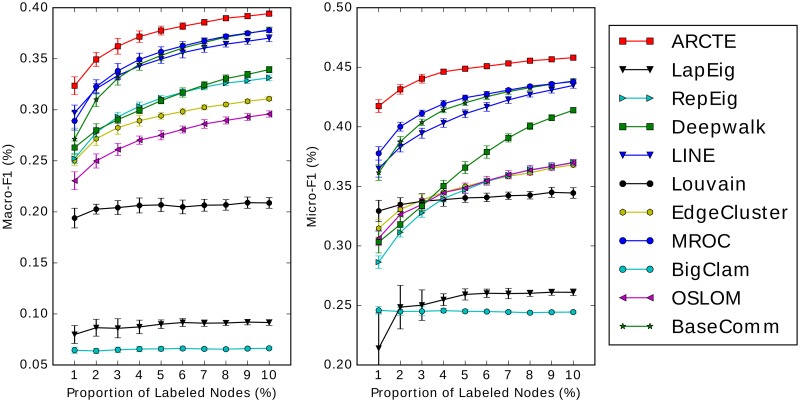
Performance on ASU-YouTube (best viewed in color).

**Fig 14:**
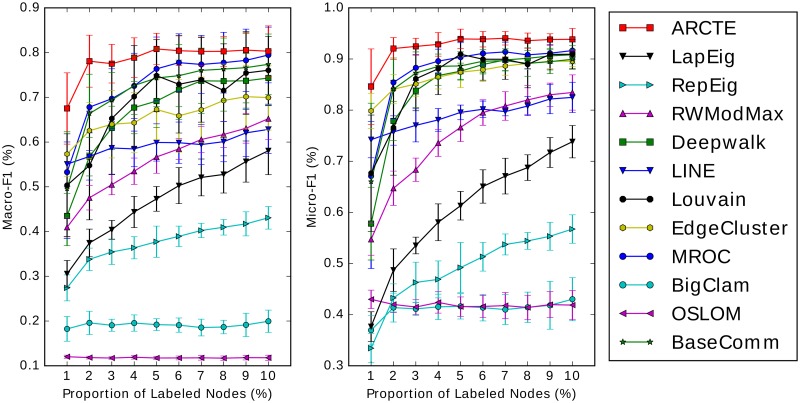
Performance on IRMV-PoliticsUK (best viewed in color).

The results for the SNOW2014G, ASU-Flickr, ASU-YouTube datasets and IRMV-PoliticsUK are depicted in Figs 11, 12, 13 and 14 respectively. In Table 6 we show for all cases depicted in Figs 11–14 the winner and runner-up methods. We further report the training set percentages for which the improvement of the highest ranked method over the second highest is statistically significant (*p* < 0.01) as calculated via a paired t-test. A fair comparison was ensured by using common training-test set partitions for each training set percentage among the different methods. Finally, for each training set percentage we calculate the maximum absolute and relative F1 score improvement percentages of the winner method over the runner-up and we report the largest for each case.

**Table 6:** Top two performing methods comparison.

		SNOW2014G	ASU-FR	ASU-YT	IRMV-PoliticsUK
Macro-F1	Winner	ARCTE	BaseComm	ARCTE	ARCTE
	Runner-up	LINE	MROC	MROC	MROC
	*tr*_%_: *p* < 0.01	{2–10}	{3–10}	{1–10}	{3–4}
	Max Improvement Abs.	4.29%	0.74%	3.44%	14.24%
	Max Improvement Rel.	22.49%	2.61%	11.92%	26.72%
Micro-F1	Winner	ARCTE	ARCTE	ARCTE	ARCTE
	Runner-up	LapEig	MROC	MROC	MROC
	*tr*_%_: *p* < 0.01	{2–10}	{1–10}	{1–10}	{3–9}
	Max Improvement Abs.	6.34%	1.43%	3.97%	17.49%
	Max Improvement Rel.	24.13%	4.59%	10.51%	26.06%

We report the inclusive training set percentages (*tr*_%_) for which there is a significant difference between the paired trial sets with *p* < 0.01 and the max absolute and relative improvement percentage. In the Macro-F1 case on ASU-Flickr ARCTE is ranked third, though it is ranked first for 1% training set with *p* < 0.01.

Firstly, we note that we used our improved cumulative PageRank differences method (Alg 7) for the similarity calculation in the ARCTE algorithm. Furthermore, we applied supervised community weighting as described in sub-section *Supervised community weighting* to all community detection methods in order to improve the results.

According to the results, ARCTE outperforms all the competing methods, with the exception of the F1-Macro measure for ASU-Flickr, where it is still competitive with respect to the winners (MROC and BaseComm) as the score difference is not significant for all training set percentages. Specifically, BaseComm surpasses ARCTE with *p* < 0.01 for training set percentages in {4–10} and ARCTE is actually the leading method with *p* < 0.01 for a 1% training set. Furthermore, ARCTE clearly surpasses BaseComm and MROC in F1-Micro. We note that for the three other datasets, ARCTE dominates by reaching even 4.29% above the runner-up for SNOW2014G, 3.44% for ASU-YouTube and 14.24% for IRMV-PoliticsUK in terms of Macro-F1 score. The maximum relative improvements reach 22.49%, 11.92% and 26.72% for the three datasets respectively. Finally, the winning performance of ARCTE is also significant with *p* < 0.01 for the majority of cases (except for the F1-Macro on ASU-Flickr) as can be seen in Table 6. Another observation of note is that ARCTE performs comparatively well for small training set percentages, as is mostly evident on ASU-Flickr, ASU-YouTube and IRMV-PoliticsUK and less so on SNOW2014G since the improvement of ARCTE gets larger for bigger percentages. As for ASU-Flickr, ARCTE surpasses MROC and BaseComm even in the Macro-F1 measure for 1% training set.

We also note that while ARCTE is consistently near the top of the competition, no other competing method can boast a similar behavior. Indeed, while Deepwalk, LINE and LapEig score quite well on SNOW2014G, albeit quite lower than ARCTE, their performance is not competitive in the other datasets. Similarly, the performance of MROC on SNOW2014G is not comparable to the rest of the competition. ARCTE also outperforms two methods based on neural word representation learning, Deepwalk and LINE, in all datasets. We chose as representatives of state-of-the-art community detection techniques the following: Louvain, OSLOM and BigClam. Generally, they are shown not to perform competitively to either low-rank matrix representations or specifically crafted community detection techniques for user classification. Indeed, the methods that are distinguished by their results are ARCTE, MROC and to a lesser extent the BaseComm approach. The latter technique requires far lower computational cost compared to the rest and is easy to implement but its performance deteriorates significantly in sparsely labeled datasets, such as SNOW2014G. Finally, although ARCTE wins the competition, MROC is a notable competitor, achieving high F1 scores for all datasets, although its behavior seems very similar to BaseComm and its quadratic complexity makes it impractical for very large graphs.

We also performed the same experiments on these datasets for a subset of the better performing methods for training set percentages up to 90%. The figures and discussion can be found in S1 Fig.

#### Feature extraction method execution times

As an empirical benchmark, we report execution times in seconds for all the competing methods in Table 7. There is an implicit caveat though: as mentioned before, we used some optimized implementations available online (e.g., BigClam) or utilized linear algebra packages (e.g., LapEig and RepEig). Our own Python implementations of ARCTE and MROC are without any effort at any noteworthy software optimization techniques, with the exception of simple coarse-grained parallelism in ARCTE. Finally, the OSLOM times reported correspond to all the clean-up runs (we noticed improved accuracy for more runs). The results from Table 7 indicate that apart from accomplishing a good performance in terms of accuracy for user classification, ARCTE is also one of the fastest methods.

**Table 7:** Feature extraction method execution times (in seconds).

Methods	Implementation	SNOW2014G	ASU-FR	ASU-YT	IRMV-PoliticsUK
ARCTE	Python	1,562	94	5,059	5
LapEig	Python/ARPACK	27,601	732	34,284	1
RepEig	Python/ARPACK	3,056	2,075	10,234	1
RWModMax	Matlab/ARPACK	✘	95,092	✘	3
Deepwalk	Python	7,010	1,615	25,151	5
LINE	C++	8,030	6,863	11,650	4,453
EdgeCluster	Matlab/C++	25	355	377	3
MROC	Python	29,263	23,748	104,954	2
BigClam	C++/openmp	181	353	274	2
OSLOM	C++	26,945	243,246	157,029	19
BaseComm	Python	1	1	1	1

### Summary of results

We now discuss five key findings based on our experimental results.

#### ARCTE parameter selection

With ARCTE, we see that as one decreases the restart probability *ρ* and the threshold parameter *ϵ*, the performance further improves with the caveat of increased execution time. By increasing them, the performance drops as it becomes more difficult to capture extended vertex-centric communities. However, even if no extended vertex-centric communities are captured, ARCTE behaves similar to the Base Communities algorithm, which performs reasonably well across datasets. This behavior, in addition to ARCTE’s robustness with respect to SVM hardness *C* perturbation attest that ARCTE is a reliable approach to user classification.

#### Cumulative PageRank differences

The promising results reported in sub-section *Similarity vector comparison* led us to an interesting observation. User-centric community detection via thresholding the PageRank vector has been used extensively in problems such as local community detection methods [34], matrix sparsification [33] and nearly linear time solution of symmetric, diagonally dominant systems [29]. Our improvement may seamlessly substitute the PageRank calculation in any of these applications, leading to faster and improved results.

#### Feature weighting for low-rank matrix representations

During the experimental design, we attempted to use feature weighting/selection also for the low-rank matrix representation methods. However, the continuous valued nature of the features produced by such methods requires additional computational steps. For example, one might try to first discretize the features and then apply feature weighting as normal. We tried discretization via bidimensional histograms along with *χ*^2^ weighting in order to weigh or remove weak features, but we did not notice any improvement; in fact there was a decrease in performance in some cases. We did not try advanced approaches, such as Parzen window discretization or numerical integration, as this would increase execution time significantly and introduce more tunable parameters, thus defeating the purpose of using a light-weight method to boost the importance of potent features.

#### Community detection for graph embedding

As was shown in section *Results*, ARCTE is a very appealing feature extraction approach for user classification. In most cases it outperforms all the competing methods and is consistently at least of comparable performance. This is especially apparent in the larger datasets (i.e. SNOW2014G, ASU-YouTube) where it significantly exceeds the performance of the main competitors, i.e. MROC, Deepwalk, LINE and LapEig. The comparison with LapEig on the ASU-YouTube dataset was missing from many recent studies [16, 18, 19, 23] that utilized the dataset. It further exhibits robustness to parameter perturbation and as a community detection method it lends itself to simple, but very effective supervised community feature weighting. The latter enables us to avoid placing confidence in features/communities for which we do not have significant evidence of their predictive power. Another way to view this is as a kind of automatic matrix dimensionality determination, which has been mentioned as an open problem for user classification before [16]. Since features that are independent of a label’s presence are weighted by 0, they are practically discarded for the specific experiment.

#### Insights on user classification comparative study

Among the community detection methods, ARCTE, MROC and BaseComm were significantly better performing than EdgeCluster, BigClam and OSLOM. We attribute this gap in performance to the fact that the former three also focus on high-resolution structure in the graph, whereas the latter three are designed to produce a mesoscopic representation of a graph. We further believe that the leading performance of ARCTE compared to the community-based methods in the majority of cases is due to the fact that it focuses on utilizing multiple resolution user-centric communities, thus providing more informative representations for each user. The better performance of ARCTE compared to all the low-rank matrix representation methods can also be attributed to the aforementioned idea, as well as to the improvement brought by the supervised community weighting. This combination allows for the exploitation of both the graph structure and the known label similarities within communities, for all community-based methods. The recent LINE and DeepWalk exhibited similar performances in all the datasets. By adopting the parameter selection of the original articles we found that DeepWalk outperforms LINE on the ASU-YouTube dataset, although LINE performs better on our own SNOW2014G dataset. LINE is the most recent method included in the comparison. The idea behind LINE is to preserve graph similarities, based on both first and second order connections in the graph. We believe that this concept is not as sound as ARCTE’s ability to capture extended user-centric communities, which may extend beyond or even exclude second order connected vertices in a principled way, i.e. based on the regularized commute-times similarity measure.

## Conclusion

In this study we leveraged the predictive potential of user-centric communities in Online Social Network user graphs for multilabel user classification. Our framework combines: *a*) the strengths of user-centric community detection for capturing local graph structure from the point of view of each user, *b*) an improvement of user-centric PageRank calculation that is tailored to local graph exploration and community detection and *c*) a supervised computational step that boosts community features based on their predictive potential. We compared our user classification framework against several state-of-the-art methods for graph-based feature extraction by applying them on a series of OSN user graph datasets. We have additionally introduced a new graph dataset for user classification.

Whereas our community weighting method can be applied to features produced by any community detection method, we saw after extensive comparisons that ARCTE, a community detection method tailored to the user classification problem, clearly outperforms methods that aim to a mesoscopic representation of a graph. The comparison was also against a number of spectral low-rank matrix representation methods, plus a recent deep representation method. Although some of these methods fared better than the baseline community detection methods, they were still exceeded by ARCTE in the majority of cases. Furthermore, ARCTE’s robustness with respect to parameter perturbation both in the feature extraction and model training steps is another reason for its success.

An additional contribution of this study is the improvement of user-centric PageRank calculation by the removal of self-loops in the random walk and the approximation of cumulative PageRank differences. This leads to a mathematically equivalent similarity vector, although with fewer iterations than existing methods, something that is crucial in our case; i.e. when we want to calculate accurate PageRank approximations for *all* vertices in a graph.

It should be noted that there is significant space for future research. We would like to assess our method’s effectiveness in identifying other kinds of behaviours, such as spam accounts based on the proximity of spam accounts in the graph due to link-farming practices [37], [36] and to investigate possible ways of further improving classification accuracy, e.g. via the seamless integration of other information modalities, such as text.

## Supporting information

S1 Appendix SNOW 2014 graph dataset preprocessing. (PDF)

S2 Appendix Lazy user-centric PageRank. (PDF)

S3 Appendix Fast user-centric cumulative PageRank differences. (PDF)

S1 Table Notation summary tables. (PDF)

S1 Fig User classification performance for extended training set. (PDF)
