# Supplementary material for: Multilabel user classification using the community structure of online networks
Source: PLoS One. 2017 Mar 9;12(3):e0173347. doi: 10.1371/journal.pone.0173347 (PMC5344401; doi:10.1371/journal.pone.0173347)
Supplement: S1 Appendix — (PDF) [file pone.0173347.s001.pdf]

S1 Appendix. SNOW 2014 Graph Dataset Preprocessing. We extracted a mention and a retweet graph from the tweet collection [1] as follows: If a tweet has a mention or is a reply, we add a directed link from the poster to the mentioned/replied-to user in the mention graph A_m. Furthermore, if the tweet is a retweet, we add a directed link from the poster to the original tweet's author in the retweet graph A_r. Finally, if we have not processed the original tweet already, we connect accordingly the original tweet's author to the mentioned/replied-to users.

We extracted the largest connected component (LCC) of the graph that is described by the directed addition of the mention and retweet adjacency matrices as calculated by $A = (A_m + A_m^{'} + A_r + A_r^{'})/4$, where $A^{'}$ denotes the transpose of matrix A. Other methods for integrating multiple graph views exist [2] and for extracting an undirected implicit graph from a directed graph [3, 4] but we have found in our experiments that they usually lead to worse performance for all methods when compared to the simple method we described in this paragraph.

As for the labelling, we selected the top 13,000 users in the summed graph in terms of PageRank and used the Twitter Search API (https://dev.twitter.com/rest/public/search) to fetch up to 500 Twitter lists for each of them. 108 of these users were suspended at the time of collection and no lists were available. We extracted a set of keywords from each list name and description and reduced these sets to associate each user with a bag of keywords. The keyword set is formed by performing a text cleaning procedure on the list description and name that is described later. We aggregated all the bags of keywords and we inspected the keywords. We manually selected labels of interest and merged several keywords under certain labels in cases where simple edit distance was not enough to capture the similarity. We formed the user-to-label frequency matrix. We performed augmented tf-idf weighting [5] to account for users being associated with disproportionately numerous labels. We associate each user with labels with tf-idf scores higher than the user-specific 80th percentile. Finally, if any label has fewer than 30 associated users, we discard the label. We ended up with 10,992 labeled users.

Text cleaning process includes the following: *a*) tokenize the concatenated Twitter list name and description, *b*) separate camel-case, *c*) use a Parts-of-Speech tagger and keep only nouns and adjectives, *d*) remove digits, punctuation and whitespace, *e*) remove stop words, *f*) perform lemmatization using the Python nltk (http://www.nltk.org/) Wordnet lemmatizer and return the set of distinct lemmas.

This labelling procedure is inspired by a recent crowd-sourcing, topical expert identification method [6]. We opted for tf-idf and percentile based thresholding in order to penalize common labels and extract labels even for niche-interest characteristics.

References

1. Papadopoulos S, Corney D, Aiello LM. SNOW 2014 Data Challenge: Assessing the Performance of News Topic Detection Methods in Social Media. In: SNOW-DC@ WWW. Seoul, Korea; 2014. p. 1–8.

2. Zhou D, Burges CJ. Spectral clustering and transductive learning with multiple views. In: Proceedings of the 24th international conference on Machine learning. Corvallis, OR, USA: ACM; 2007. p. 1159–1166.

3. Chung F. Laplacians and the Cheeger inequality for directed graphs. Annals of Combinatorics. 2005;9(1):1–19.

4. Lai D, Lu H, Nardini C. Finding communities in directed networks by pagerank random walk induced network embedding. Physica A Statistical Mechanics and its Applications. 2010;389(12):2443–2454.

5. Manning CD, Raghavan P, Schütze H. Introduction to information retrieval. vol. 1. Cambridge university press Cambridge; 2008.

6. Ghosh S, Sharma N, Benevenuto F, Ganguly N, Gummadi K. Cognos: crowdsourcing search for topic experts in microblogs. In: Proceedings of the 35th international ACM SIGIR conference on Research and development in information retrieval. Portland, OR, USA: ACM; 2012. p. 575–590.
