# Supplementary material for: Multilabel user classification using the community structure of online networks
Source: PLoS One. 2017 Mar 9;12(3):e0173347. doi: 10.1371/journal.pone.0173347 (PMC5344401; doi:10.1371/journal.pone.0173347)
Supplement: S2 Appendix — (PDF) [file pone.0173347.s002.pdf]

S2 Appendix. Lazy User-centric PageRank. In order to elaborate on our first improvement, we consider the lazy Markov chain transition matrix $W_\lambda = \lambda I + (1-\lambda)D^{-1}A$, where we call λ the *laziness factor*, i.e. the probability that the agent performs a self-loop at any step t. The motivation behind the addition of self-loops [1] with probability $\lambda = {}^1/_2$ was their leading to a faster distribution convergence and the reinforcement of vertices near the seed by slowing probability diffusion. This idea was adopted in the fast PageRank method by [2]. *We generalize the system by parameterizing by λ to show that this is an unnecessary and misleading artifact in the calculation of the user-centric PageRank vector.*

Due to λ, the single element PageRank and residual update rules are altered as in Eqs 12, 13. Laziness implies self-loops in W and hence probability equal to $(1-\rho)\lambda r^{(t)}(u)$ remains on $r^{(t)}(u)$ after each iteration. If this probability is more than $\varepsilon d(u)$, an additional number of iterations on the same vertex are needed until ${}^{r(u)}/_{d(u)} < \varepsilon$.

$$k_{\lambda pr}^{(t+1)} = k_{\lambda pr}^{(t)} + r^{(t)} I_u \tag{12}$$

$$r^{(t+1)} = r^{(t)} - \lambda r^{(t)} I_u + (1-\lambda)(1-\rho) W_{u:} r^{(t)}, \tag{13}$$

At this point, we argue that these additional iterations can be omitted by considering the application of a very large number of updates on element u such that $r(u) \to 0$. The probability that would be pushed from $r(u)$ to $k_{\lambda pr}(u)$ equals to the limit of the geometric sequence in Eq 14. As we have mentioned before, we set the initial residual distribution to be equal to e_v, for uniformity. This is why we multiply the updates of Eqs 3 (in the main article) and 12 by a factor of ρ, which is also found in the calculation of the limit in Eq 14. Similarly, the probability from $r(u)$ that is distributed to u's neighbors is described in Eq 15.

$$\sum_{t=0}^{\infty} \rho r^{(t)}(u)(1-\rho)^t \lambda^t = \frac{\rho r^{(t)}(u)}{1-\lambda(1-\rho)} = \rho_{eff} r^{(t)}(u) \tag{14}$$

$$\sum_{t=0}^{\infty} r^{(t)}(u)(1-\lambda)(1-\rho)^{t+1} \lambda^t = \frac{(1-\lambda)(1-\rho) r^{(t)}(u)}{1-\lambda(1-\rho)} = (1-\rho_{eff}) r^{(t)}(u), \tag{15}$$

One might have arrived at the same result by considering the steady-state solution $k_{pr}^{(t+1)} = k_{pr}^{(t)} = k_{pr}^{(\infty)}$ of the PageRank definition, described in Eq 16.

$$k_{pr}^{(\infty)} = \rho e_v + (1-\rho) k_{pr}^{(\infty)} W_\lambda \tag{16}$$

$$= \rho e_v + (1-\rho) k_{pr}^{(\infty)} (\lambda I + (1-\lambda) W) \tag{17}$$

$$= \frac{\rho}{1-\lambda(1-\rho)} e_v + \frac{(1-\lambda)(1-\rho)}{1-\lambda(1-\rho)} k_{pr}^{(\infty)} W \tag{18}$$

$$= \rho_{eff} e_v + (1-\rho_{eff}) k_{pr}^{(\infty)} W, \tag{19}$$

We have shown a statement, parameterized by λ, that clearly exposes the *distinction* between regular and lazy PageRank. Indeed, the introduction of laziness in approximate user-centric PageRank is *misleading*, as it implies a *different* PageRank vector with a λ-dependent effective restart probability $\rho_{eff} = \frac{\rho}{1-\lambda(1-\rho)}$.

Given that the laziness factor leads to a different PageRank result that is reachable through non-lazy approximate methods (that obviate unnecessary computational costs by exploiting the observations in Eqs 14 - 16), we opt to remove it from the approximate PageRank calculation. The computational gain is made by the fact that *only one iteration* is required on u for satisfying the threshold ${}^{r(u)}/_{d(u)} < \varepsilon$.

References

1. Spielman DA, Teng SH. A local clustering algorithm for massive graphs and its application to nearly-linear time graph partitioning. arXiv preprint arXiv:08093232. 2008;.
2. Andersen R, Chung F, Lang K. Local graph partitioning using pagerank vectors. In: Foundations of Computer Science, 2006. FOCS'06. 47th Annual IEEE Symposium on. Berkeley, CA, USA: IEEE; 2006. p. 475–486.
