# Supplementary material for: Multilabel user classification using the community structure of online networks
Source: PLoS One. 2017 Mar 9;12(3):e0173347. doi: 10.1371/journal.pone.0173347 (PMC5344401; doi:10.1371/journal.pone.0173347)
Supplement: S3 Appendix — (PDF) [file pone.0173347.s003.pdf]

S3 Appendix. Fast User-centric Cumulative PageRank Differences. We now derive the incremental rule for calculating $ck_{\delta pr}$ in Eq 20.

$$\begin{aligned} ck_{\delta pr}^{(t+1)} &= ck_{\delta pr}^{(t)} + \rho e_v + (1-\rho)k_{pr}^{(t)}W - \rho e_v - (1-\rho)k_{pr}^{(t-1)}W \\ &= ck_{\delta pr}^{(t)} + (1-\rho)(k_{pr}^{(t)} - k_{pr}^{(t-1)})W = ck_{\delta pr}^{(t)} + (1-\rho)r^{(t-1)}I_u W \\ &= ck_{\delta pr}^{(t)} + (1-\rho)r^{(t-1)}W_u, \end{aligned} \tag{20}$$

The importance of this derivation lies in the fact that the cumulative PageRank difference vector is always one *full* global random step ahead of PageRank, for the same number of iterations. This is equal to multiple single element updates/iterations; which is important due to the overhead at each iteration. Furthermore, when the process is repeated for all graph vertices, all available information that can be extracted per iteration is of great importance. This improvement is achieved because the quantity used in the update $(1-\rho)r^{(t-1)}W_u$ is *already* calculated during the residual update. The residual update is common among the two methods, requiring $d(u)$ multiplications and $d(u)+1$ addition assignments. We also note that the computations required for Eq 6 (in the main article) differ from Eq 3 (in the main article) *only* by having $d(u)$ addition assigments instead of 1, since the second term in Eq 6 (in the main article) is calculated once for the update of the residual (Eq 4 in the main article) and reused for updating the similarity vector. In practice, this translates to a minimal overhead, since we can leverage the same loop as the one for the residual update or parallel vectorized assignment.

We now show how our variation is also a valid similarity measure by considering that a similarity vector k seeded from vertex v is an approximation of the v-th row of a matrix K. According to Eqs 2 and 5 in the main article we get the Random-Walk-with-Restart (RWR) [1] similarity matrix in both cases. We use $\sum_{t=0}^{\infty} A^t = (I-A)^{-1}$ for a sub-stochastic matrix A.

$$\begin{aligned} K_{pr}^{(\infty)} &= \rho I + (1-\rho)WK_{pr}^{(\infty)} \\ &= \rho(I-(1-\rho)W)^{-1} \quad = \rho K_{RWR} \end{aligned} \tag{21}$$

$$\begin{aligned} K_{\delta pr}^{(\infty)} &= I + \textstyle\sum_{T\leftarrow 1}^{\infty}(1-\rho)WK_{\delta pr}^{(T)} \\ &= I + \textstyle\sum_{T\leftarrow 1}^{\infty}(1-\rho)^T W^T I \\ &= I + (I-(1-\rho)W)^{-1} - I \quad = K_{RWR}, \end{aligned} \tag{22}$$

When using PageRank approximations for user-centric community detection, the distribution is often multiplied by D^{-1} in order to reinforce proximity-based relatedness, by penalizing large degree vertices which tend to attract random walk based importance. The result is also known as regularized commute-times [2] and it holds that $K_{RCT} = K_{RWR}D^{-1}$. We will also adopt this step since the RCT kernel has been shown by [3] and [4] to outperform RWR.

References

1. Pan JY, Yang HJ, Faloutsos C, Duygulu P. Automatic multimedia cross-modal correlation discovery. In: Proceedings of the tenth ACM SIGKDD international conference on Knowledge discovery and data mining. Seattle, WA, USA: ACM; 2004. p. 653–658.

2. Zhou D, Bousquet O, Lal TN, Weston J, Schölkopf B. Learning with local and global consistency. Advances in neural information processing systems. 2004;16(16):321–328.
3. Fouss F, Francoisse K, Yen L, Pirotte A, Saerens M. An experimental investigation of kernels on graphs for collaborative recommendation and semisupervised classification. Neural Networks. 2012;31:53–72.
4. Papadimitriou A, Symeonidis P, Manolopoulos Y. Fast and accurate link prediction in social networking systems. Journal of Systems and Software. 2012;85(9):2119–2132.
