# Supplementary material for: Multilabel user classification using the community structure of online networks
Source: PLoS One. 2017 Mar 9;12(3):e0173347. doi: 10.1371/journal.pone.0173347 (PMC5344401; doi:10.1371/journal.pone.0173347)
Supplement: S1 Table — (PDF) [file pone.0173347.s004.pdf]

S1 Table. Notation Summary Tables.

Summaries for the notation-heavy sections *Graph-based User Classification* and *Proposed Framework*, that contain the symbols necessary to comprehend the Algorithms 1-8 (in the main article), can be found in Tables 8 and 9.

Table 8. Notation Summary - *Graph-based User Classification.*

Symbols	Definitions
$G = (V, E)$	graph (i.e. network of users)
V	set of vertices (i.e. users)
E	set of edges (i.e. social relations)
A	graph's adjacency matrix
$a_{u,v} = A(u, v)$	edge weight from user u to v $\forall u, v \in E$
$A_{u:}, A_{:v}$	single row or column of matrix A
$N(v), d(v)$	set of neighbor vertices and degree of vertex v
L	set of available labels that characterize users
V_l, V_u	labeled and unlabeled user sets
y_v	labeling vector for user $v \in V_l$, $y_v(l) = \begin{cases} 1, & \text{if } v \text{ labeled as l} \in L \\ 0, & otherwise \end{cases}$
Y	label matrix, where each row $Y_{v:} = y_v$
Y_l, Y_u	label matrices corresponding to sets V_l and V_u
X	user coordinate (i.e. feature) matrix in latent space
X_l, X_u	feature matrices corresponding to sets V_l and V_u
dim	latent space dimensionality
h	hypothesis that maps user projections $X_{v:}$ to label vectors $Y_{v:}$
C	the full set of communities detected by a method
$\|C\|$	the number of communities
c	the set of vertices belonging to a community
$\|c\|$	the number of vertices in a community
$b_v = N(v) \cup v$	the base vertex-centric community of vertex v
e_v	the extended vertex-centric community of vertex v

Table 9. Notation Summary - *Proposed Framework*.

Symbols	Definitions
k	sparse similarity vector (ARCTE definition)
nnz(k)	the non-zero element indices of k
W	Markov chain transition probability matrix
k_{rw}	similarity vector based on random walk
ρ	restart probability
ρ_{eff}	effective restart probability
k_{pr}	PageRank similarity vector (i.e. random walk with restart)
λ	laziness factor - probability of agent not transitioning to another vertex
$k_{\lambda pr}$	lazy PageRank similarity vector
$ck_{\delta pr}$	cumulative PageRank differences similarity vector
r	residual probability distribution
ε	residual probability threshold
./	elementwise vector division operation
$d_{ave}(v)$	average degree of neighborhood $N(v)$
$d_{max}(v)$	maximum degree of neighborhood $N(v)$
$d_{min}(v)$	minimum degree of neighborhood $N(v)$
$ivf(j)$	inverse vertex frequency for community/dimension j - unsupervised
M	contingency matrix
$PSNR(M)$	function that calculates peak noise-to-signal ratio for all rows of M
$\chi^2(j)$	weight based on chi squared test for community/dimension j - supervised
