# Supplementary material for: Multilabel user classification using the community structure of online networks
Source: PLoS One. 2017 Mar 9;12(3):e0173347. doi: 10.1371/journal.pone.0173347 (PMC5344401; doi:10.1371/journal.pone.0173347)
Supplement: S1 Fig — (PDF) [file pone.0173347.s005.pdf]

S1 Figure. User Classification Performance for Extended Training Set.
For three of our four datasets (SNOW2014G, ASU-Flickr, ASU-YouTube), we executed the top performing methods for an additional number of training set ratios: [15%, 20%, 25%, 30%, 40%, 50%, 60%, 70%, 80%, 90%] using the parameters from Table 4 of the article. For the dataset IRMV-PoliticsUK the additional training percentages are [60%, 70%, 80%, 90%] since we had already reported the performance until the 50% training percentage. We executed the experiments only for those methods that exhibited a competitive performance across all the datasets, namely: ARCTE, LINE, Deepwalk, LapEig, MROC and BaseComm.

The results for the four datasets for these additional training percentages are shown in Figures 15-18.

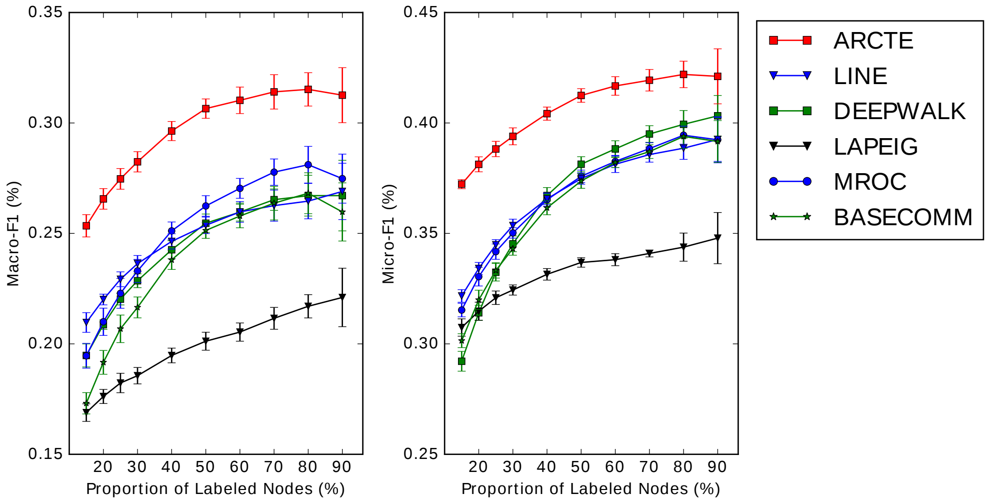

Fig 15. Performance on SNOW2014G for additional training percentages (best viewed in color).

From what we can see in the Figures 15 and 18, the performance of ARCTE relative to the competing methods for the SNOW2014G and IRMV-PoliticsUK datasets remains similar compared to the 1%-10% percentage range. Specifically, ARCTE is a clear leader in the former case and also achieves a competitive performance in the latter by being in a tie with MROC in F1-Macro and leading in F1-Micro.

There are some differences in the cases of ASU-Flickr and ASU-YouTube, shown in Figures 16 and 17. Specifically, for ASU-Flickr, although ARCTE still remains the top performing method for F1-Micro, in the case of F1-Macro it exhibits an improvement compared to the other methods by *surpassing* the MROC method for percentages larger than 50%. On the other hand, even though ARCTE was by far the top performing method for the ASU-YouTube dataset in the 1% - 10% percentages, the extended experiments have shown us something different. At the 50% mark, the BaseComm method surpasses ARCTE for F1-Macro, whereas for F1-Micro the LINE and BaseComm methods surpass ARCTE at the 30% and 50% marks respectively. However, ARCTE is by far the one leading in the smaller percentages, in what is arguably the most difficult range of the experiment due to the sparse annotation. We believe that this is the result of the user-centric community approach to feature extraction. On a closing

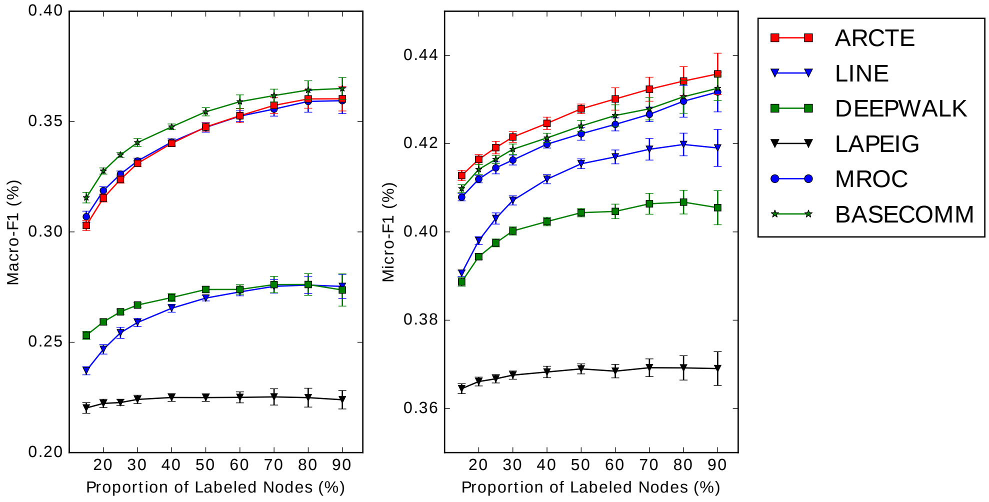

Fig 16. Performance on ASU-Flickr for additional training percentages (best viewed in color).

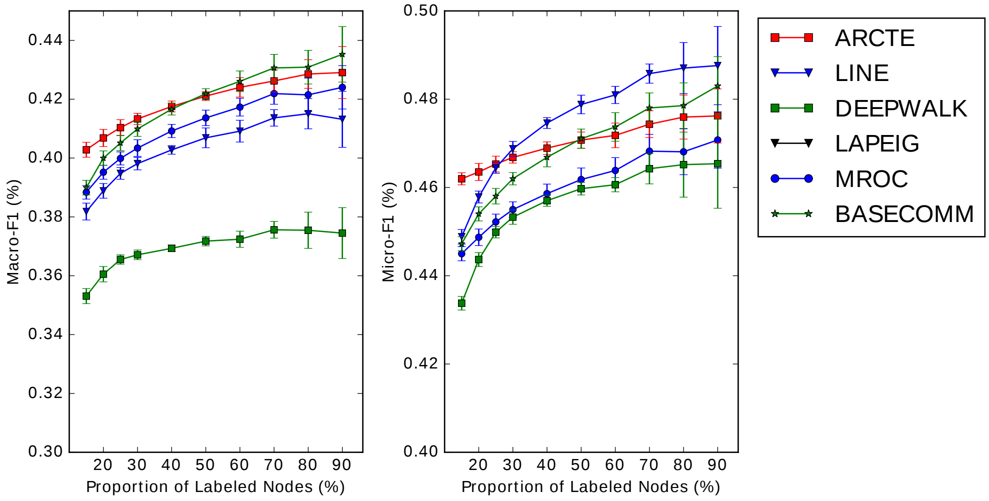

Fig 17. Performance on ASU-YouTube for additional training percentages (best viewed in color).

note, the fact remains that the competing methods' performance varies significantly in the other datasets, whereas ARCTE is consistently at the top or very near to it.

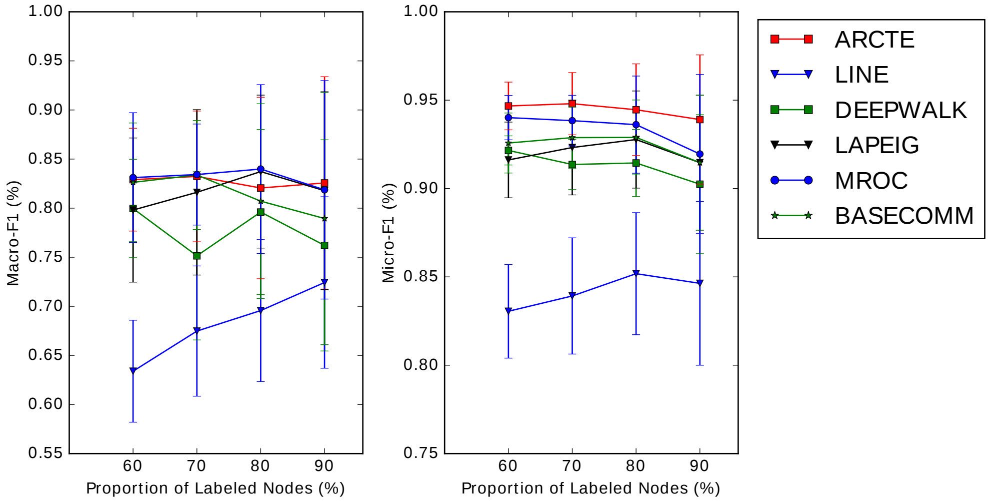

Fig 18. Performance on IRMV-PoliticsUK for additional training percentages (best viewed in color).
